# Misclassified: identification of zoonotic transition biomarker candidates for influenza A viruses using deep neural network

**DOI:** 10.3389/fgene.2023.1145166

**Published:** 2023-07-27

**Authors:** Nissrine Hatibi, Maude Dumont-Lagacé, Zakaria Alouani, Rachid El Fatimy, Mounia Abik, Tariq Daouda

**Affiliations:** ^1^ Ecole Nationale Supérieure d'Informatique et d'Analyse des Systèmes, Mohammed V University in Rabat, Rabat, Morocco; ^2^ Institute of Biological Sciences (ISSB), UM6P Faculty of Medical Sciences, Mohammed VI Polytechnic University, Ben Guerir, Morocco; ^3^ Piercing Star Technologies, Montreal, QC, Canada

**Keywords:** neural networks, virus, influenza, zoonotic transition biomarkers, sequencing

## Abstract

**Introduction:** Zoonotic transition of Influenza A viruses is the cause of epidemics with high rates of morbidity and mortality. Predicting which viral strains are likely to transition from their genetic sequence could help in the prevention and response against these zoonotic strains. We hypothesized that features predictive of viral hosts could be leveraged to identify biomarkers of zoonotic viral transition.

**Methods:** We trained deep learning models to predict viral hosts based on the virus mRNA or protein sequences. Our multi-host dataset contained 848,630 unique nucleotide sequences obtained from the NCBI Influenza Virus and Influenza Research Databases. Each sequence, representing one gene from one viral strain, was classified into one of the three host categories: Avian, Human, and Swine. Trained models were analyzed using various neural network interpretation methods to identify interesting candidates for zoonotic transition biomarkers.

**Results:** Using mRNA sequences as input led to higher prediction accuracies than amino acids, suggesting that the codon sequence contains information relevant to viral hosts that is lost during protein translation. UMAP visualization of the latent space of our classifiers showed that viral sequences clustered according to their host of origin. Interestingly, sequences from pandemic zoonotic viral strains localized at the margins between hosts, while zoonotic sequences incapable of Human-to-Human transmission localized with non-zoonotic viruses from the same host. In addition, host prediction for pandemic zoonotic sequences had low prediction accuracy, which was not the case for the other zoonotic strains. This supports our hypothesis that ambiguously predicted viral sequences bear features associated with cross-species infectivity. Finally, we compared misclassified sequences to well-classified ones to extract interesting candidates for zoonotic transition biomarkers. While features varied significantly between pairs of species and viral genes, several codons were conserved in Swine-to-Human and Avian-to-Human misclassified sequences, and in particular in the NA, HA, and NP genes, suggesting their importance for zoonosis in Humans.

**Discussion:** Analysis of viral sequences using neural network interpretation approaches revealed important genetic differences between zoonotic viruses with pandemic potential, compared to non-zoonotic viral strains or zoonotic viruses incapable of Human-to-Human transmission.

## 1 Introduction

Influenza is one of the most common zoonotic viral infections that affects both Humans and animals. Although most influenza infections are from annual seasonal epidemics, sporadic global pandemic outbreaks also occur involving influenza A virus strains of zoonotic origin. Pandemic influenza is characterized by the introduction of a new strain of influenza A virus for which there is no pre-existing immunity in Humans, as the new strain is antigenically different from previously circulating strains. This lack of pre-existing immunity is often associated with increased infection severity, and an increase in mortality (Krammer et al., 2018).

Identifying biomarkers that are predictive of viral hosts, whether from surface protein or from other structural and functional viral genes, is of interest to identify features that could play a role in zoonotic viral transition. Influenza A viruses circulate not only in Humans but also in domestic animals, pigs, horses and poultry and in wild migratory birds. Viral hosts are usually identified using empirical evidence derivation methods such as laboratory testing, surveillance, and other epidemiological evidence, including phylogenetic analysis. However, bioinformatics tools have been incorporated into influenza research in recent years to improve our understanding of interspecies transmission. Computational approaches are now playing an important role, with the deployment of novel methodologies combining bioinformatics, machine learning and deep learning approaches to predict emerging zoonotic viral strains. Several research groups have searched for host-specific markers across the entire influenza A virus genome. Various bioinformatics methods based on multiple sequence alignment (Chen et al., 2006; Finkelstein et al., 2007; Allen et al., 2009; Miotto et al., 2010), information theory (Sjaugi et al., 2015), and combinatorial modeling (Khaliq et al., 2016) have also been used to identify specific amino acid residues or motifs within viral proteins that differentiate between Avian and Human viruses. Others have attempted to apply machine-learning approaches to build computational models to predict Avian-to-Human transmission of influenza A viruses directly using protein sequences (Qiang and Kou, 2010; Wang et al., 2013a; Wang et al., 2013b).

Deep neural networks have a significant advantage over other machine learning methods for sequence classification, as they can extract relevant and complex classification features from genetic sequences without prior knowledge. Deep neural networks have already demonstrated outstanding results in the analysis of viral sequences; Mohamed et al. (2021) proposed an approach for predicting sequences using the seq2seq LSTM neural network considering sequences as text data, for accurate and fast prediction of mutations of RNA viruses in the development of antiviral drug resistance. The effectiveness of their proposed model was established against the Influenza Virus Dataset and the New Castle Disease Database, with 98.9% and 96.9% accuracy, respectively. Their results illustrate the potential of LSTM neural networks for solving sequence analysis issues in bioinformatics. Mock et al. (2021) constructed a deep neural network to predict viral hosts for three different virus species based on viral genome sequences only. Their model achieved a very high accuracy with AUC ranging between 0.94 and 0.98.

In this work, we analyzed 848,630 unique nucleotide sequences of Influenza A viruses extracted from the NCBI Influenza Virus (Schoch et al., 2020) and Influenza Research Databases (Zhang et al., 2017) in search of zoonotic transition biomarker candidates that could be used to predict which viral strains are most likely to transition between Avian, Swine and Humans. We elected to use state-of-the-art Natural Language Processing algorithms that have been previously applied with great success to biological sequence analyses: Bidirectional LSTMs (Hochreiter and Schmidhuber, 1997), and Transformers (Vaswani et al., 2017). We first built host classification models capable of classifying Influenza A viral mRNA and protein sequences using Bidirectional LSTM and Transformers. Both types of Deep Learning models showed greater accuracy when trained on mRNA sequences, rather than protein sequences, suggesting that mRNA sequences contain information relevant to predict viral host that is lost during protein translation. We then tested the hypothesis that sequences that are difficult to classify should bear features that are typically associated with other species, and thus could be the best candidates for zoonotic transition makers. We evaluated how zoonotic viruses were classified by our models and whether zoonotic viruses capable of Human-to-Human transmission would be differentiated by our model from zoonotic viruses that are not capable of Human-to-Human transmission. Results showed that sequences of zoonotic viruses capable of Human-to-Human transmission are ambiguous to our model and behave very differently compared to non-zoonotic viruses and zoonotic viruses that are not capable of Human-to-Human transmission. These results confirmed that our model was able to detect sequences of zoonotic strains with pandemic potential and supported our hypothesis that these pandemic strains presented features that made them ambiguous to the network. Finally, we analyzed sequences that were misclassified by our best model using statistical tests (Student’s t-test, Fisher’s Exact test), information theory (Kullback-Leibler divergence), and machine learning interpretation methods (LIME (Zhang et al., 2019)) to extract features associated with these misclassified sequences that represent interesting candidates for zoonotic transition biomarkers in Influenza A viruses.

## 2 Materials and methods

### 2.1 Data source and preprocessing

Data was obtained from the NCBI Influenza Virus Database, which contains the sequences of all influenza A viruses in the EMBL/DDBJ/GenBank databases (Schoch et al., 2020), and Influenza Research Database (FLU DB, https://legacy.fludb.org/brc/home.spg?decorator=influenza) (Zhang et al., 2017). Each sequence represents one gene from one viral strain. Duplicated sequences, i.e., that were found in both databases, were removed to keep unique sequences only. The combined dataset contained 848,630 unique nucleotide sequences, with 264,579 Avian, 467,415 Human, and 116,636 Swine sequences (Supplementary Figure S1A).

As the number of sequences per host was not balanced, an under-sampling strategy was used to ensure that the networks were presented with the same number of examples for each host at each training epoch. The protein dataset was obtained by translating nucleotide sequences. Models were trained on 70% of the dataset, 15% were reserved for the validation set and 15% for the test set. 116,636 sequences per host were kept, for a total of 349,908 sequences used for training, validation and testing of the models (Supplementary Figure S1B).

To make sequences digestible to deep learning algorithms, mRNA and protein sequences were further processed by associating a unique index to every codon and amino acid (indexes of 1–20 for amino acids and 1–61 for codons, stop codons being removed, see Supplementary Figure S2A). Input size was fixed to the maximum sequence length of 770 codons or amino-acids (from the PB2 gene) and zero padding at the end of the gene sequences was used to ensure that all input sequences have the same length (Supplementary Figure S2B). Finally, hosts were encoded with a unique identifier (Supplementary Figure S2C).

### 2.2 Prediction models

Two different models were trained to predict hosts from mRNA or protein sequences (Supplementary Figure S3). The architecture of the first model consists of an embedding layer followed by a Bidirectional LSTM layer. Bidirectional LSTMs are recurrent neural networks reading the sequence from both ends to identify relevant patterns (Hochreiter and Schmidhuber, 1997). Their recurrent aspect allows them to handle both long and short-term dependencies in sequences. The LSTM layer is followed by a dropout layer to prevent overfitting, then two dense fully connected layers for integrating the output of the LSTM. Each layer consists of 100 units, with the exception of the output layer, which has three units, one for each species.

The second architecture also starts with an embedding layer, followed by a Transformer layer that generates a vector for each time step of the input sequence, followed by a dropout layer, then two dense and dropout layers, each layer consisting of 100 units, and finally a Softmax output layer with three units. In contrast to the first network, Transformers use attention mechanisms to identify relevant patterns in sequences (Vaswani et al., 2017). All models were built with the Python package Keras using the Tensorflow back-end.

Hyperparameters were optimized using random sampling over: Batch size, Number of layers and Size of layers. The best architecture was selected on the validation set and final results were reported on the separate test set. Number of epochs were optimized using early-stopping on the validation set. For everything else, including layer parameter initialization and Adam optimizer we used the default values of Keras version 2.12.0. The best results were obtained after 16 epochs for the Bidirectional LSTM, and 20 epochs for Transformers. Hyperparameters were optimized on the validation set (Supplementary Table S1). Host class imbalance was handled using an under-sampling strategy, ensuring that models were trained using the same number of examples for each host. Networks were trained using the categorical cross-entropy loss and the Adam optimizer. Host prediction accuracy is reported for the test set.

### 2.3 Predictions on zoonotic virus sequences

To assess their performance in predicting viral hosts, a total of 584 zoonotic viral sequences were analyzed using trained models. Importantly, these zoonotic sequences were not included in the training, nor the validation set (see Supplementary Tables S2, S3 for information and accession numbers of zoonotic sequences analyzed). Zoonotic sequences included in Supplementary Table S2 were identified following a direct transmission from Avian-to-Human (*n* = 23) or Swine-to-Human (*n* = 85) without subsequent Human-to-Human transmission (Subbarao et al., 1998; Garten et al., 2009; Shinde et al., 2009; Schoch et al., 2020). Sequences in Supplementary Table S3 are all from the Swine 2009 pandemic influenza strain (*n* = 476), thus originating from Swine but with the capacity for Human-to-Human transmission (Garten et al., 2009). Predicted viral hosts for these zoonotic mRNA sequences as classified by the Bidirectional LSTM model were extracted. Then, UMAP (McInnes et al., 2018) was used to visualize the zoonotic sequences in the previous latent space. The centroid of each cluster was obtained using the K-means algorithm on the UMAP output. Finally, the Euclidean distance was calculated (using the UMAP dimensions) between each sequence and each host cluster centroid.

Euclidean distances for each set of zoonotic sequences was compared to those of well-classified non-zoonotic viruses from each host class using a Kruskal–Wallis test, followed by a *post hoc* Dunn test. Adjusted *p* values from the Dunn test are reported.

Proportions of well-classified and misclassified sequences in each zoonotic subset were compared using Fisher’s exact test.

### 2.4 Extracting features in misclassified sequences with UMAP

After selecting the best models for both mRNA and protein sequences, we extracted misclassified sequences for further analyses. For this analysis, we used a combination of the test set and of all sequences discarded during undersampling. Thus, a total of 598,414 sequences are included in the statistical evaluation of features associated with misclassified sequences. If the prediction of the viral host is the same as the ground truth, the sequence is considered well-classified. Misclassified sequences were then compared to well-classified sequences to determine features that make them different. We use the notation < ground-truth-host>_to_<predicted-host > to denote misclassified sequences, e.g., Avian-to-Human refer to Avian viral sequences that were predicted to be virus from Human host. Misclassified sequences are always compared to the well-classified from their ground truth host, e.g., Avian-to-Human misclassified sequences (i.e., sequences from strains from Avian hosts classified by the network as strains from Human hosts) will be compared to well-classified Avian sequences.

We used two statistical tests to identify biomarkers that are significantly enriched or depleted in misclassified sequences: Student’s t-test, used to measure the differences between the means of two groups, and Fisher’s exact test, used to determine if there are nonrandom associations between two categorical variables. In addition, we used the Kullback-Leibler divergence to measure the divergence in distribution of features between misclassified and well-classified sequences.

Finally, we used LIME (Zhang et al., 2019) to understand the impact of specific features on the predictions of our models. This approach was used in the analysis of zoonotic transition biomarkers in overall sequences and within specific genes. LIME works by modulating the input to the model, by randomly modifying a specific input to the network and monitoring the impact on the predictions to determine how specific features influence predictions. In the context of sequence classification, this consists in randomly replacing or masking codons or amino acids to determine which ones influence the prediction the most.

Finally, results of the four aforementioned methods were combined to calculate a consensus score of zoonotic transition features. When a feature is identified as significantly different by one of the four methods, it is attributed a score of 1. When it is identified as significantly different by three of the four methods, it is attributed a score of 3, and so on. The sign of the score (+ or −) is then assigned depending on whether the feature is enriched (more frequent) in misclassified sequences (+) or depleted (less frequent) in misclassified sequences (−) compared to well-classified ones.

## 3 Results

### 3.1 Using mRNA instead of protein sequences as input increases prediction accuracy

We first investigated which of mRNA or protein sequences were more informative in predicting viral host using artificial neural networks. Bidirectional LSTM and Transformers models were tested in parallel for both types of input datasets. Both models achieved a higher accuracy when receiving mRNA sequences as inputs (Table 1; Supplementary Table S4). Proportions of well-classified and misclassified sequences for each model, type of input and host are shown in Supplementary Table S5. These results suggest that mRNA sequences contain information relevant to the prediction of viral host that is lost during translation in proteins.

**TABLE 1:** Accuracy of host classification models.

	Bidirectional LSTM	Transformers
mRNA	0.9565	0.9511
Proteins	0.9333	0.9352

### 3.2 Misclassified and zoonotic viral sequences localize at the margins of host clusters

**FIGURE 1:**
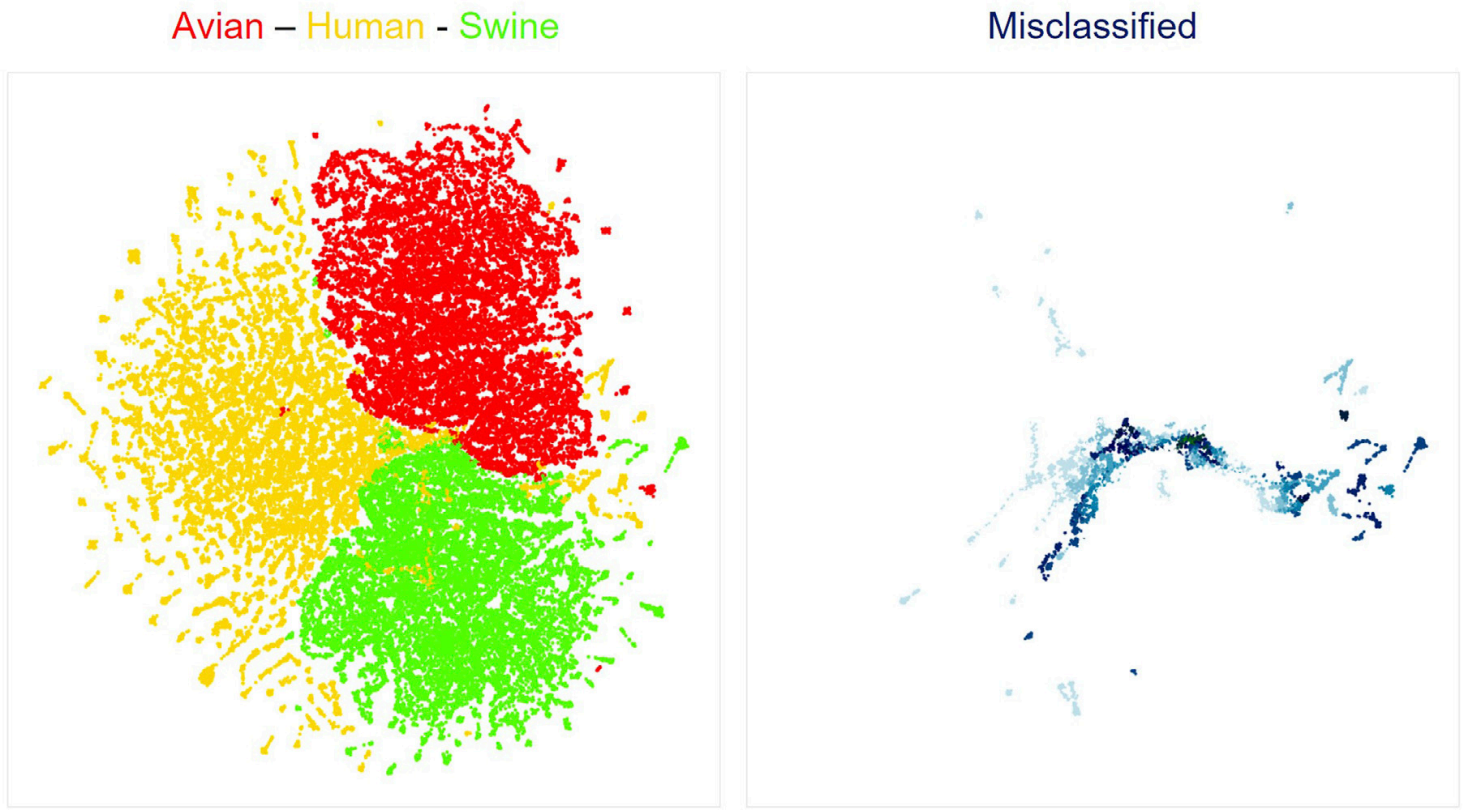
UMAP projection of latent spaces of the Bi-LTSM model trained on mRNA sequences. On the left is shown the UMAP visualization of well-classified sequences by hosts (red: Avian, yellow: Human, green: Swine). On the right is shown the UMAP visualization of misclassified sequences in the same latent space. Opacity shows the degree of model inaccuracy. The darker the color, the more inaccurate the prediction, meaning that the network assigned a larger probability to an incorrect class. Each dot represents one gene from one viral strain.

We next used neural network interpretation methods to extract relevant biological information from viral sequences. We first visualized the latent space of our classifiers using the UMAP algorithm (McInnes et al., 2018). As expected from the high accuracies of all models, sequences are clearly separated by hosts (Figure 1, left; Supplementary Figure S4A). Interestingly, sequences that were misclassified by the models were generally localized at the margins between different host clusters (Figure 1, right; Supplementary Figure S4B). Of note, UMAP projections compress a complex dimensional space in a 2D space. Thus, the true margins between clusters of sequences have a more complex shape than shown in Figure 1, as the original latent space has 7 dimensions. Nonetheless, these visualizations are helpful to build an intuitive understanding of how deep learning networks represent viral sequences.

Because of the high accuracy obtained on host prediction, we hypothesize that the features learned by the network are highly indicative of the host infected by the strain. We further reasoned that a sequence that is ambiguous to the model, i.e., is located at the margin of a class or misclassified by the network, likely bears features of strains associated with more than one host and thus represent interesting candidates for zoonotic transition biomarkers.

To test this hypothesis, we assessed whether sequences from zoonotic viral strains would locate at the margins between hosts and/or would be misclassified by the network. We analyzed two different sets of zoonotic viral sequences: one containing 108 sequences (85 sequences from Swine and 23 from Avian) from zoonosis originating from a direct Swine-to-Human or Avian-to-Human transition (Supplementary Table S2), and one containing 476 unique sequences from the 2009 pandemic H1N1 strains (Supplementary Table S3). These two sets of viruses differ in that the first set contains viruses that were not capable of Human-to-Human transition, while the 2009 H1N1 strains did. Of note, these sequences were not in the training or validation sets.

**FIGURE 2:**
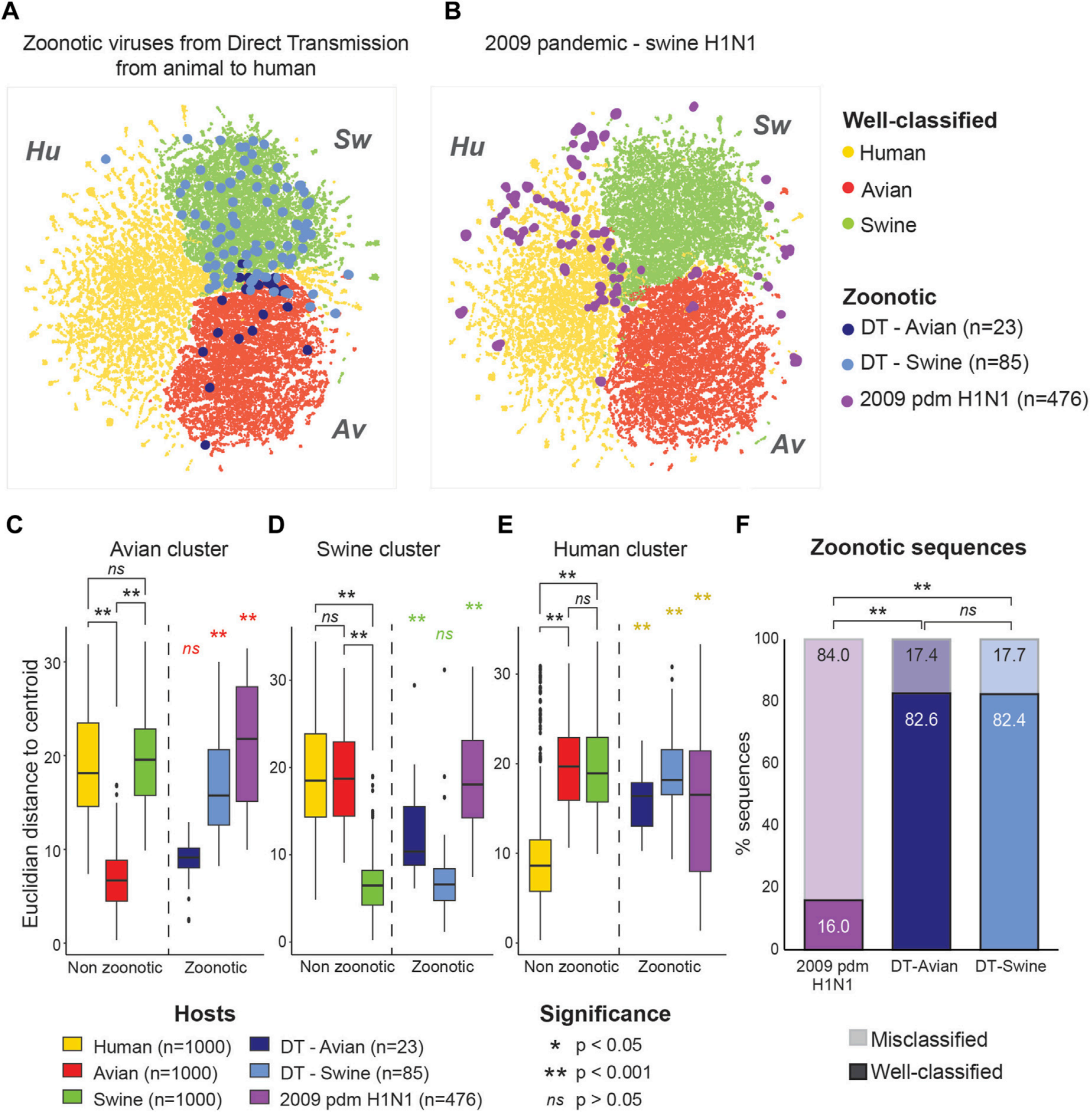
Zoonotic sequences of pandemic potential are located at the margins of host clusters. Localization of zoonotic sequences in the model’s latent space is projected over well-classified non-zoonotic viral sequences for the three different hosts (Avian: red, Human: yellow and Swine: green). **(A)** Sequences from zoonotic virus derived from direct animal-to-Human transmission (DT) are shown according to their host of origin (Avian, n = 23: dark blue, Swine, n = 85: light blue). **(B)** Sequences from the 2009 pandemic H1N1 Swine Influenza A (purple, n = 476). **(C–E)** Euclidean distances from the clusters’ centroid for each group of viral sequences. Distance from the Avian **(C)**, Swine **(D)** and Human **(E)** clusters are shown separately. Distribution of distances were compared using Kruskal–Wallis test for nonparametric distributions, followed by a *post hoc* Dunn test for pair comparisons. Euclidean distances for the well-classified viral sequences were calculated on a randomly selected subset of 1,000 sequences per host. **(F)** Proportions of well-classified and misclassified sequences in the zoonotic virus subsets. Percentage of well-classified sequences are shown with full colors (lower bars), while the percentage of misclassified sequences is shown with transparency (upper bars). Significance is assessed using Fisher exact test.

To evaluate whether these sequences are ambiguous to the model, we extracted their coordinates within the latent space of the model (Bidirectional LSTM trained on mRNA) and projected them using the same UMAP projection. As shown in Figure 2A, sequences from zoonotic viruses from direct contact with an animal tended to localize within their original host clusters. In contrast, pandemic zoonotic sequences from the 2009 H1N1 Swine Influenza located at the margins of the Swine and Human cluster (Figure 2B). These results show that the model is able to detect genetic features that are unique to viral strains of pandemic potential, different from both non-zoonotic viruses and zoonotic viruses without Human adaptation.

To formally compare the positioning of zoonotic sequences in the latent space, we trained a K-means algorithm to determine the centroid of each host cluster. We then calculated Euclidean distances between each sequence and the centroid of each host cluster. We compared the distances of zoonotic sequences to the cluster centroids to that of well-classified viral sequences from non-zoonotic viruses of each host. Zoonotic viruses derived from direct transmission were treated separately according to their host of origin.

Zoonotic viruses derived from direct transmission showed the same distribution of distance from the centroid of their host cluster as well-classified (non-zoonotic) viruses of the same host (see Figure 2C for direct transmission from Avian viruses; Figure 2D for direct transmission from Swine viruses). In contrast, the sequences from the Swine pandemic H1N1 strains behaved very differently, being significantly further from the centroids of all host clusters compared to non-zoonotic viruses (Figures 2C–E). This pattern was found in all genes separately (Supplementary Figure S5). This is in line with results from Garten et al. (2009) which showed that the 2009 Swine pandemic strains did not have molecular markers for Human adaptation and is coherent with the fact that the sequences from the 2009 pandemic H1N1 strains do not overlap the Human virus cluster either (Figures 2B, E).

We also compared the proportion of well-classified and misclassified sequences in each subsets of zoonotic viral sequences. While only 17.4% and 17.7% of sequences of zoonotic viruses from direct transmission were misclassified by the model, 84.0% of the Swine 2009 pandemic H1N1 sequences were misclassified (Figure 2F). These results show that both the localization in the latent space and the prediction of the model are informative as to the pandemic potential of the viral sequence.

Taken together, these results show that zoonotic sequences localize with misclassified sequences at the margins between the different host classes. This distinct localization of sequences from zoonotic strains of pandemic potential far from their host cluster, along with the lower host prediction accuracy of the model, reveals that the network was able to capture genetic features that are key to cross-species infectivity.

### 3.3 Extracting features of misclassified sequences as candidates for zoonotic transition markers

Zoonotic sequences and viral sequences misclassified by our models tend to concentrate into the marginal space between hosts (Figures 1, 2). This suggests that the neural networks have identified signals, or unique features within viral sequences that could render the viruses more likely to cross species boundaries. These signals constitute interesting candidates for zoonotic transition biomarkers. Therefore, to identify and characterize these features and understand what makes them ambiguous to the networks, we compared the misclassified sequences to well-classified ones. As prediction results obtained with the Bidirectional LSTM were superior to those obtained with the Transformers, we elected to use this network for the fine-grained analysis of mRNA sequences.

To identify candidates for zoonotic transition markers, we first assess codon usage on viral sequences by comparing misclassified sequences to well-classified ones for each pair of species. We elected to combine four different methods to detect significant enrichment in codon usage: (1) Student’s t-test, (2), Fisher’s exact test, (3), Kullback Leibler and (4) LIME (see Section 2 for more details). We then calculated a score for each codon which reflects the number of methods that identified this codon as significantly different between well-classified and misclassified sequences (between 0 and 4). We assigned a positive (+) or negative (−) sign to the score to represent whether a codon was enriched or depleted, respectively, in misclassified sequences compared to well-classified ones. For instance, a given feature significantly enriched in misclassified sequences according to two methods will be attributed the score +2.

**FIGURE 3:**
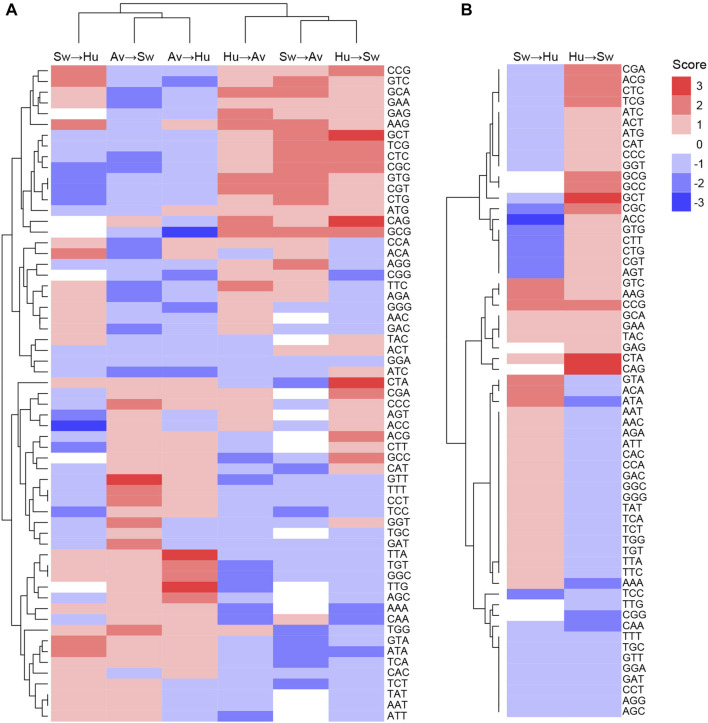
Codons likely to enhance or reduce zoonotic transition for each pair of species. Codon scores were clustered for **(A)** all pairs of species, or **(B)** for Human and Swine pairs, in both transition directions. Scores represent the number of methods which identified a codon as significantly enriched (positive sign, shown in red) or depleted (negative sign, shown in blue) in misclassified sequences.

Codons showing differential usage between well-classified and misclassified sequences are shown in Figure 3A (details for each codon and each pair of species can be found in Supplementary Table S6). Several codons appear to enhance or to reduce zoonotic transitions. For instance, AAG(Lys), ACA(Thr), ATA(Ile), CCG(Pro), GTA(Val), and GTC(Val) have a score of +2 in Swine-to-Human transition, which suggests that they could enhance zoonotic transition from Swine to Human (Figure 3A, third column). In contrast, ACC(Thr), AGT(Ser), CGC(Arg), CGT(Arg), CTG(Leu), CTT(Leu), GTG(Val) and TCC(Ser) have a score of −2 or −3 in Swine-to-Human transition, suggesting that it reduces the likelihood of zoonotic transition in this pair of species. Of note, several codons identified as having differential usage between well-classified and misclassified sequences are synonymous codons (e.g., Glycine codons GGC and GGG have scores of +2 and −2 for Avian-to-Human sequences, respectively, or Threonine codons ACC and ACA have scores of −3 and +2 for Swine-to-Human, respectively), suggesting that codon usage is likely contributing to the capacity of a virus to transition from one species to the other (Supplementary Table S6). Best candidates for Swine-to-Human and Avian-to-Human zoonotic transition biomarkers are listed in Table 2.

**TABLE 2:** Best candidates for zoonotic transition markers in Swine-to-Human and Avian-to-Human misclassified sequences.

Swine-to-Human		Avian-to-Human	
Score	Codons	Score	Codons
+3	—	+3	TTA, TTG
+2	ATA, AAG, CCG, ACA, GTA, GTC	+2	TGT, GGC, AGC
−2	AGT, CGC, CTG, CTT, GTG, TCC	−2	CGG, GGG, ATC, GTC
−3	ACC	−3	GCG

Interestingly, patterns of codons that appear to enhance or reduce transition are very similar for the Avian-to-Human and Avian-to-Swine pairs, as well as for the Human-to-Avian and Swine-to-Avian pairs, as shown by their clustering in Figure 3A. This suggests that similar codon features could be associated with zoonotic transition from Avian to any of the other two species and *vice versa*.

Interestingly, some codons appear to have inverse effects in a given pair of species. For instance, CGC(Arg) has a score of −2 for Swine-to-Human transition and +2 for Human-to-Swine transition, while ATA(Ile) has a +2 score for Swine-to-Human and −2 for Human-to-Swine transition (Figure 3B). This inverse relationship suggests that CGC(Arg) is better suited for viral infections in Human, while ATA(Ile) would be better suited for Swine. Some codons appear to favor zoonotic transitions in one direction only (e.g., CAG(Gln) has a score of 0 in Swine-to-Human transition, but +3 for Human-to-Swine transition). Results for other species pairs can be found in Supplementary Figure S6; Supplementary Table S6. Taken together, these results suggest an important degree of complexity in how codon usage affects the likelihood of a virus to transition from one species to another.

### 3.4 Zoonotic transition biomarkers differ by gene and by position

**FIGURE 4:**
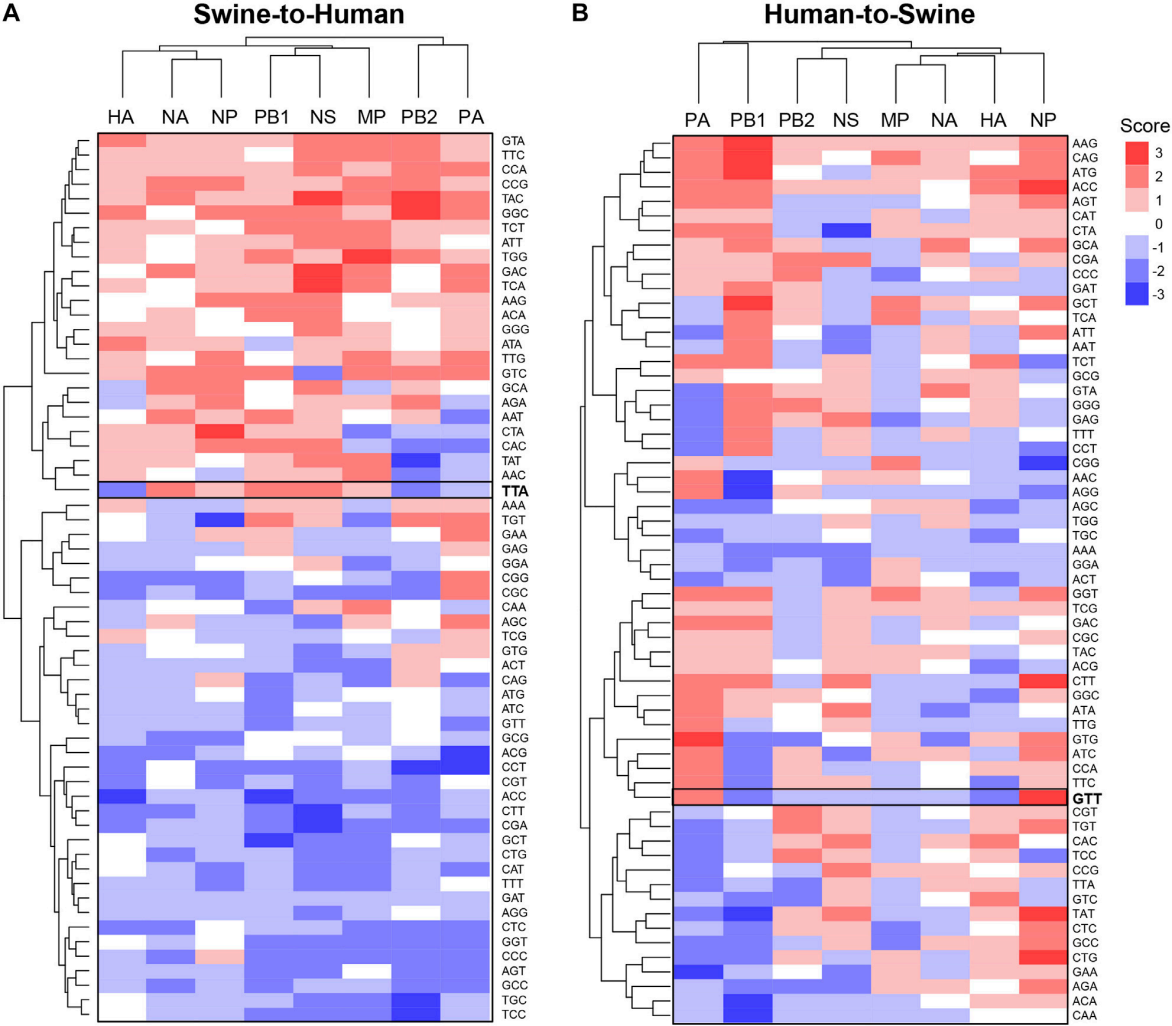
Candidate zoonotic transition biomarkers differ between viral genes. Codons’ scores were clustered for **(A)** Swine-to-Human, or **(B)** for Human-to-Swine misclassified sequences. Scores represent the number of methods which identified each codon as significantly enriched (positive sign) or depleted (negative sign) in misclassified sequences.

Using the same approach, we further analyzed codon features of misclassified sequences within specific genes. Interestingly, codons identified as significantly enriched or depleted for a given pair of species are different between genes (Figure 4 for Swine-Human pairs, see Supplementary Figure S7 for Avian-Human pairs). For instance, TTA(Leu) shows a score of −2 for HA and PB2 but a score of +2 for NA, PB1 and NS in Swine-to-Human misclassified sequences (highlighted in Figure 4A). Similarly, GTT(Val) shows an enrichment for NP (score of +3) and PA (+2), but a depletion for PB1 and HA (−2) in Human-to-Swine misclassified sequences (Figure 4B). Best candidates per gene for Swine-to-Human and Avian-to-Human zoonotic transition biomarkers are listed in Table 3.

**TABLE 3:** Best candidates for zoonotic transition markers for each gene in Swine-to-Human and Avian-to-Human misclassified sequences.

Gene	Swine-to-Human				Avian-to-Human			
Score	−3	−2	+2	+3	−3	−2	+2	+3
HA	ACC	ACG, CCT, CGA, CGC, CGG, CGT, CTC, CTT, TTA	ATA, GGC, GTA	—	GTC	ACC, CCT, CTC, CTT, GAT, GCT, GGT, TCC	CCG, GGC, TCA, TTG	GCC
MP	—	ACC, ACT, CAG, CAT, CCC, CGA, CGC, CTA, CTC, CTG, GCT, GGA, GGT, TCC, TGC, TGT, TTT	AAC, ATT, CAA, CCG, GAC, GTA, GTC, TAC, TAT, TCA, TCT, TTC, TTG	TGG	—	GAT, TAC	TTG	GCT
NA	—	ACG, CCC, CGG, CTC, CTG, CTT, GCC, GCG	AAT, CCG, GAC, GCA, GTC, TAC, TTA	—	CCC, GCA, GGG, TCC	AGG, CAC, CAT, CCA, CTG, GCG, GTC	ACC, ATG, CAA, CGT, GCC, GCT, GGC, TCT, TTG	—
NP	TGT	CAT, CCT, CGC, CGG, CGT, GCG, TTT	AAG, AGA, CAC, CCG, GCA, GGC, GTC, TTG	CTA	—	AAC, AGA, CAG, CCG, CGT, GAG, GTG	AAA, ATA, CTT, TCC	TTA
NS	CGA, CTT	ACC, ACT, AGC, AGG, AGT, CAT, CCC, CCT, CGC, CGT, CTG, GCT, GGT, GTC, GTG, TCC, TGC, TTT	AAG, ACA, ATT, CAC, CCA, GCA, GGC, GGG, GTA, TAT, TCT, TTA, TTC	GAC, TAC, TCA	—	CTC, CTG, GAA, GAC	AAC	—
PA	ACG, CCT	AAT, AGT, CAC, CAG, CAT, CCC, CGA, CTC, GCC, GGT, GTT	AGC, CCA, CGC, CGG, GAA, GAC, GGC, GTC, TAC, TCA, TGT, TTG	—	—	CCG, CGA, CTG, GAA, GAT, GCA, GCG, TCT, TGC	ACT, CTT, TGT, TTG	GGA
PB1	ACC, GCT	AGT, ATC, ATG, CAA, CAG, CCC, CCT, CGA, CTT, GCC, GGT, GTT, TCC	AAG, AAT, ACA, CAC, GGC, GTC, TCT, TGG, TGT, TTA	—	AGG	TAC, TTG	AGA, CTA, GGG, TTA, TTT	—
PB2	CCT, TAT, TCC, TGC	AAC, ACC, AGT, CAC, CCC, CGA, CGC, CGG, CGT, CTC, CTT, GCC, GGT, TTA	AGA, CCA, CCG, GTA, GTC, TGG, TGT, TTC	GGC, TAC	ATC, CTA, TTC	AAG, ACA, CGC	AAA, ACT, AGA, AGC	—

**FIGURE 5:**
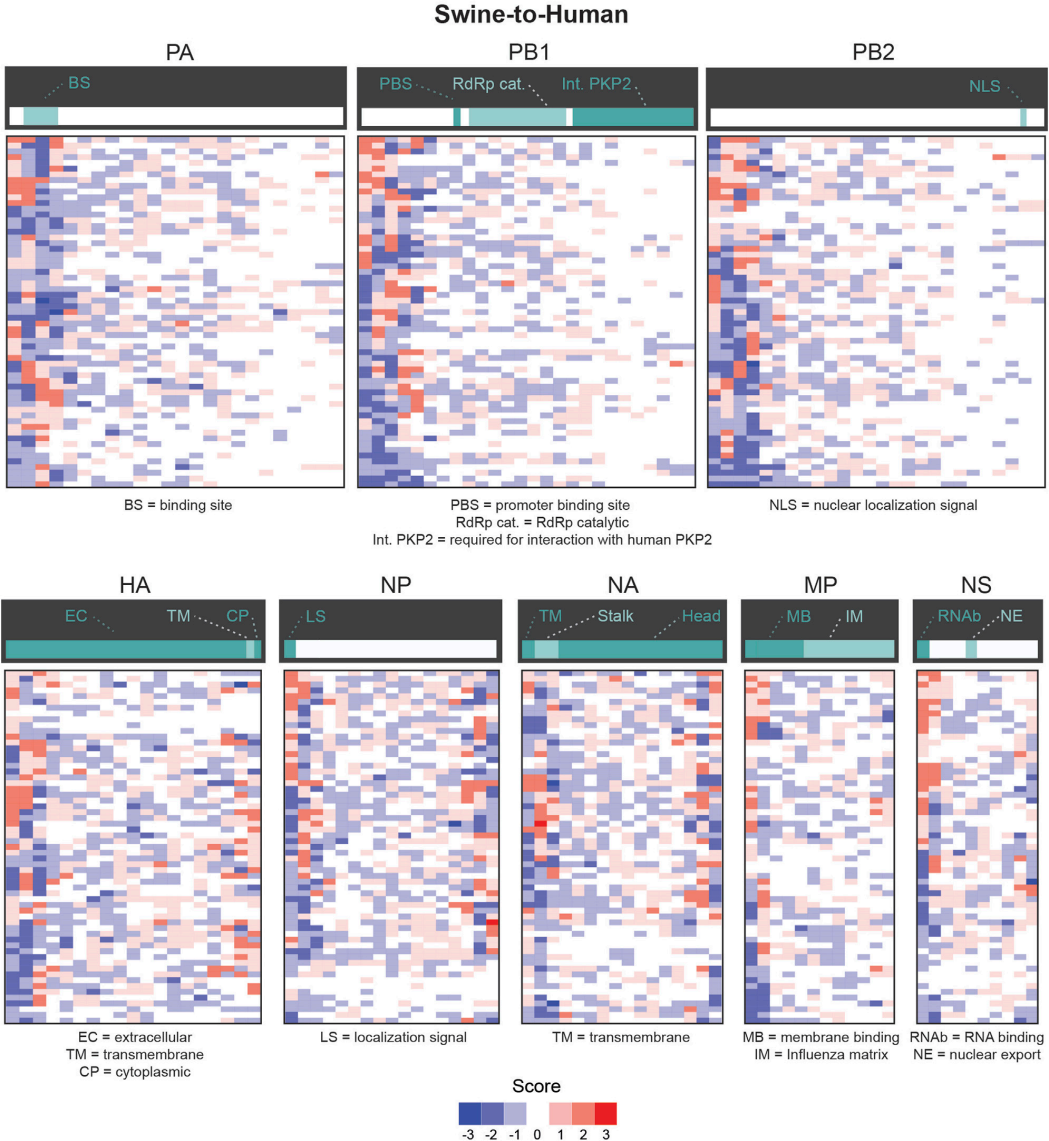
Candidate zoonotic transition biomarkers by position for Swine-to-Human misclassified sequences. Codon features analyzed separately for each section of 30 codons in each Influenza genes for for Swine-to-Human misclassified sequences. Scores represent the number of methods which identified each codon as significantly enriched (positive sign) or depleted (negative sign) in misclassified sequences. Important gene domains are shown above in green shades.

We next evaluated whether certain regions of interest (ROI) of each gene were more likely to contain significantly different features in misclassified sequences. We focused our analysis on the number of appearances of codons in slices of 30 consecutive codons. We define ROIs for a given pair (i.e., codon and gene) as the regions in that gene sequence where a codon mutation is likely to be most impactful on zoonotic transition. As shown in Figure 5, most significantly enriched or depleted features are localized at the beginning of each gene in Human-Swine pairs of species. For HA, NP and NA genes, several codons were significantly enriched or depleted at the end of the gene as well. This was also the case for the Human-Avian pairs (Supplementary Figure S8).

**FIGURE 6:**
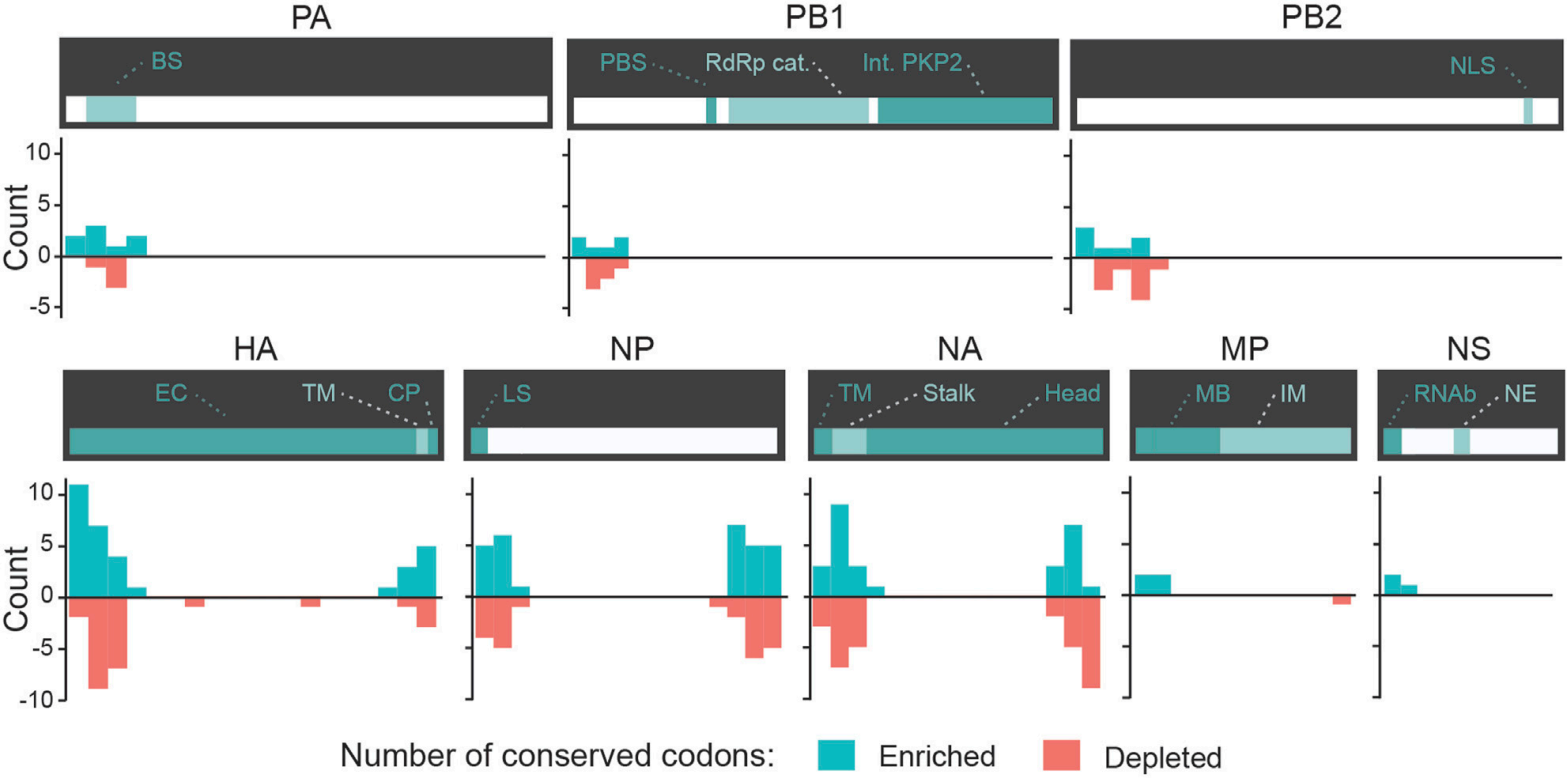
Conserved candidate zoonotic transition biomarkers by position in Swine-to-Human and Avian-to-Human misclassified sequences. Number of codons (counts) with scores ≤ −2 or ≥2 in both Avian-to-Human and Swine-to-Human for each gene region. Only codons with similar type of divergence (both ≤ −2 or both ≥2) are shown.

We evaluated whether some codons were identified as candidate biomarkers for zoonotic transition in the same ROI and with the same type of divergence (either enrichment or depletion) in both Avian-to-Human and Swine-to-Human misclassified sequences. We focused our analysis on codons with scores ≤ −2 or ≥2 Avian-to-Human and Swine-to-Human. Interestingly, HA, NA, and NP genes showed the greatest number of codons that were conserved in misclassified sequences in both Avian-to-Human and Swine-to-Human misclassified sequences, which tend to locate and the beginning and end of these three genes (Figure 6). This highlights the importance of these three genes in zoonosis in Humans. These conserved codons therefore represent the best candidates for zoonotic transition biomarkers in Humans (shown in Table 4).

**TABLE 4:** Codons conserved in Swine-to-Human and Avian-to-Human misclassified sequences per ROI.

Gene	ROI	Enriched	Depleted
HA	[0,29]	AAG, ACC, AGG, ATA, ATT, CAA, GAC, GCG, GGA, TTA, TTT	ATC, GTA
	[30,59]	AAC, AAG, ATT, CAA, CTT, GGA, TCT	ACA, ACG, CAC, CTG, GAG, GCT, GGG, GTG, TTG
	[60,89]	AAC, CGA, CTC, TCA	AAG, CCT, CGG, CTA, CTT, GAC, TGC
	[90,119]	GTG	—
	[180,209]	—	GTT
	[360,389]	—	AAA
	[480,509]	TTT	—
	[510,539]	AAC, AGT, TTG	TTT
	[540,569]	ATC, CAG, CTG, GGG, TCC	AAC, TCA, TGC
MP	[0,29]	ACA, CTG	—
	[30,59]	GGA, TGC	—
	[330,359]	—	AAT
NA	[0,29]	ACA, GGA, TTA	CTA, GTA, GTT
	[30,59]	AGC, CAA, CAC, CTT, GGA, GTA, GTT, TCA, TGC	ACC, ATA, CTG, GCT, GGG. GTG, TTC
	[60,89]	GAC, GCC, TTT	AAG, AGA, AGG, ATT, GTA
	[90,119]	TCC	—
	[390,419]	CAG, GAG, GGG	AGC, CAA
	[420,449]	AAC, ACT, CGA, CTA, GGG, GTT, TTC	ATT, GAG, GGA, TCA, TTT
	[450,479]	GAG	AAT, ACA, ATA, CAA, CTA, GAT, GGA, GGG, TTC
NP	[0,29]	ACA, CAA, GAG, GAT, GCG	AAT, CAG, GTT, TCT
	[30,59]	AGA, ATT, CTA, CTT, GAT, TGG	AGT, CAG, CAT, CTG, GAA
	[60,89]	CTT	ACA
	[390,419]	—	TCT
	[420,449]	AAC, AAT, CAG, CGA, CTC, GAC, TTC	TCA, TTT
	[450,479]	AAG, GAG, TAC, TCC, TTG	ATC, CCT, GAA, GAT, GTT, TCT
	[480,509]	AAC, AGT, GGG, TAC, TTT	AAT, GAG, GGA, TAT, TTC
NS	[0,29]	GGA, GGG	—
	[30,59]	TCA	—
PA	[0,29]	AAC, GGA	—
	[30,59]	AAT, CAA, GTT	TTC
	[60,89]	GTT	CCT, GAT, GGG
	[90,119]	ATA, GCT	—
PB1	[0,29]	GAC, TTT	—
	[30,59]	GAT	ACG, AGG, CCG
	[60,89]	GCT	CGG, TTA
	[90,119]	AGG, ATA	CAG
PB2	[0,29]	AAC, AAG, AGG	—
	[30,59]	TAC	AAA, GAA, TGT
	[60,89]	GTT	GGG
	[90,119]	ATC, GAT	ATT, GTC, TAC, TGT
	[120,149]	—	CAC

Taken together, these results underline a great degree of complexity in what might influence zoonotic transition from one species to another and suggest that zoonotic transition biomarkers are likely to differ for each gene according to their position in the sequence.

## 4 Discussion

Predicting which viral strains are likely to transition from one species to another from their genetic sequence could help in the prevention and response against these zoonotic strains. We hypothesized that features that are predictive of viral hosts could be leveraged to identify biomarkers of zoonotic transition. We first investigated the usability of Deep Learning for the prediction of hosts (Avian, Human, and Swine) from viral mRNA or protein sequences. We compared two deep learning methods, namely, Bidirectional LSTM and Transformers. In all cases, we obtained very high accuracies on both protein and mRNA sequences, with the highest accuracies obtained using mRNA sequences. This is consistent with previous results of Behura and Severson, (2013); Velazquez-Salinas et al. (2016) highlighting the importance of codon usage bias for viral evolution. These results also suggest that codons are better markers of zoonotic transition than amino acids, in accordance with previous studies (Wong et al., 2010; Fancher and Hu, 2011; Sun et al., 2020).

One of the most salient features of the model was its capacity to specifically distinguish zoonotic viral sequences from strains with pandemic potential. Indeed, pandemic zoonotic sequences from the 2009 H1N1 Swine Influenza located at the margins of the Swine and Human clusters and were mostly misclassified by the model. This was not the case for viruses extracted from a direct Swine-to-Human transmission, which did not subsequently result in Human-to-Human transmission. Interestingly, the same patterns were found for all genes separately. These results are in line with the study from Garten et al. (2009) which showed that the 2009 Swine pandemic strains did not have molecular markers for Human adaptation. These results strongly suggest that the model can detect genetic features that are unique to viral strains of pandemic potential, different from both non-zoonotic viruses and zoonotic viruses without Human adaptation.

In the second part of the study, we use Deep Neural Networks interpretation techniques to identify candidates for zoonotic transition biomarkers. More specifically, we compared sequences that were misclassified by the network to well-classified sequences using statistical tests, the Kullback-Leibler divergence and the LIME Deep Learning interpretation method (Zhang et al., 2019). We showed that candidate features vary significantly between pairs of species, sometimes appearing to have inverse effects in a given pair of species, sometimes appearing to favor transition in only one direction. We also showed that candidate features differed between different viral genes and tended to be more prominent at the beginning and end of each gene. Interestingly, several of those candidate biomarkers were the same in both Swine-to-Human and Avian-to-Human misclassified sequences, suggesting their importance for zoonosis in Humans. The number of conserved codons in these two groups of misclassified sequences were particularly high at the N- and C-terminal of NA, HA and NP genes, highlighting their crucial role in cross-species infectivity.

Our results suggest two levels of viral adaptation to the host. The first level is the global codon adaptation to the host, in accordance with previous studies (Wong et al., 2010; Fancher and Hu, 2011; Sun et al., 2020). Indeed, viruses are subjected to evolutionary pressures to adapt their mRNA sequences to their hosts. As different hosts have different codon usage biases and different tRNA pools, viruses often evolve to adapt by adapting their global codon usage to their hosts (Chen et al., 2020). Our results also suggest a second and more fine-grained level of adaptation where the position of the codon in the sequence is also important. This could be due to translation kinetics that require codons at a certain position to modulate translation efficiency and accuracy and therefore protein folding (Rodnina, 2016). Previous studies have also linked codon usage to protein misfolding and the generation of Defective Ribosomal Products (DRiPs) (Drummond and Wilke, 2008) that are rapidly degraded by the proteasomal machinery and are preferentially presented by the MHC-I antigen presentation machinery (Cannarozzi et al., 2010; Plotkin and Kudla, 2011). The usage of specific codons at certain positions that we have identified could be an evolutionary strategy to reduce the production of DRiPs.

Globally, our results support the use of Deep Learning models in the study of genetic sequences. The models used herein allow a higher resolution analysis to unlock a better understanding of the genetic nuances that can influence a given biological phenomenon. This approach is sufficiently general to be applied not only to other types of viruses, but also in other biological contexts. Because neural network models take into account more data (e.g., codon usage by position), they are also likely to be more accurate in predicting the targeted outcome and their interpretation can be leveraged to identify novel biomarkers.

## Data Availability

Publicly available datasets were analyzed in this study. This data can be found here: https://github.com/NissrineH/Misclassified
