# Supplementary material for: Misclassified: identification of zoonotic transition biomarker candidates for influenza A viruses using deep neural network
Source: Front Genet. 2023 Jul 27;14:1145166. doi: 10.3389/fgene.2023.1145166 (PMC10415530; doi:10.3389/fgene.2023.1145166)
Supplement: Supplementary file 1 [file DataSheet1.docx]

**Supplementary Material**

Misclassified: Identification of zoonotic transition biomarker candidates for Influenza using Deep Neural Network

**Nissrine Hatibi^1,2^, Maude Dumont-Lagacé^3^, Zakaria Alouani^2^, Rachid El Fatimy^2^, Mounia Abik^1^, Tariq Daouda^2^***

^1^ ENSIAS, Mohammed V University in Rabat, Morocco

^2^ Faculty of Medical Sciences, University Mohamed VI Polytechnic, Ben Guerir, Morocco

^3^ Piercing Star Technologies, Canada

*** Correspondence:**Tariq Daouda
[tariq.daouda@um6p.ma](mailto:tariq.daouda@um6p.ma)

# Supplementary Figures and Tables

## Supplementary Figures


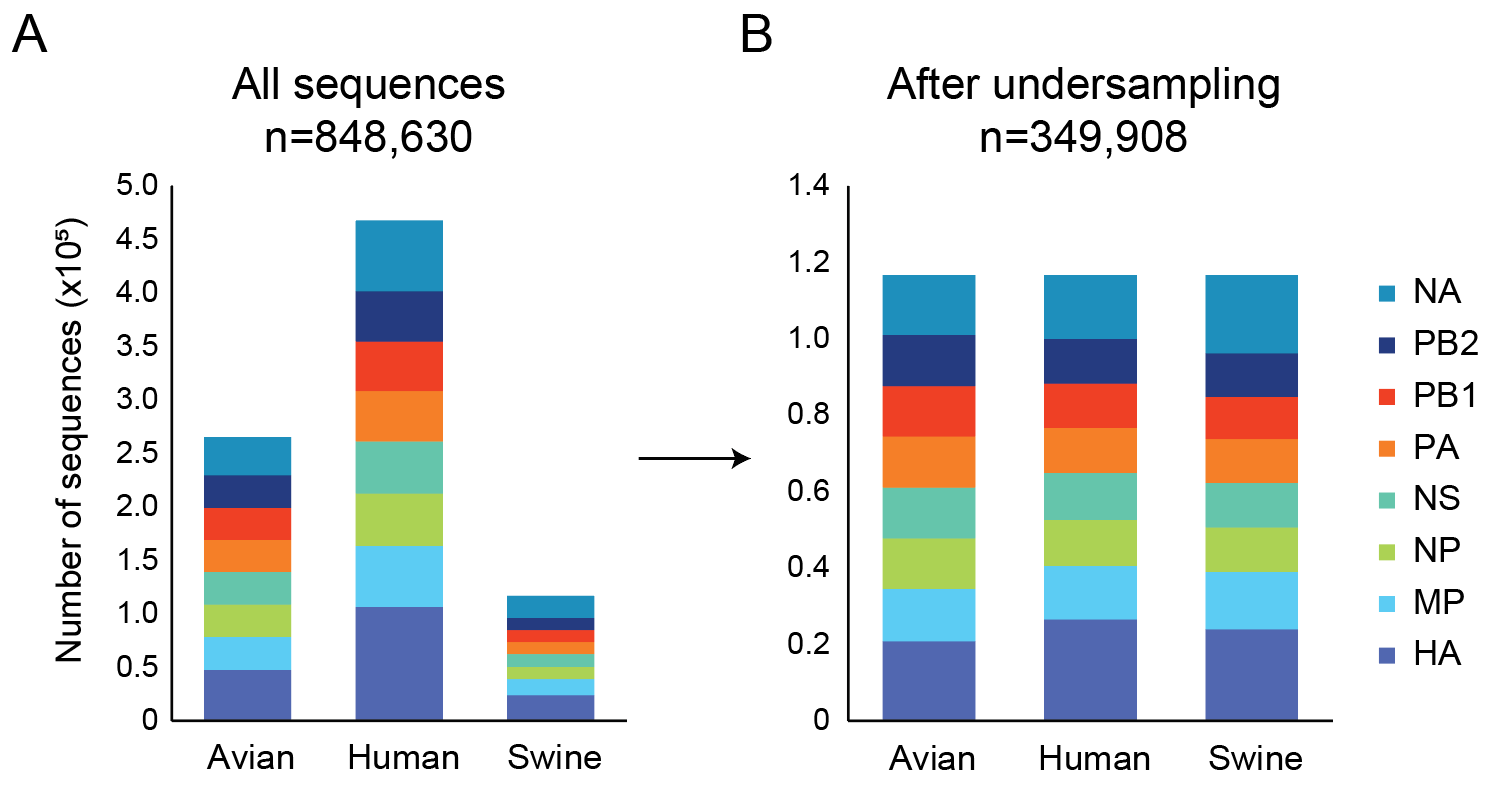


**Supplementary Figure 1. Distribution of sequences by host and gene.** (A) There were 848,630 unique nucleotide sequences extracted from the combined databases. Each sequence represents one gene from one viral strain. (B) Following under-sampling, a total of 116,636 sequences were randomly selected per host, for a total of 349,908 sequences used for train, validation and test sets.


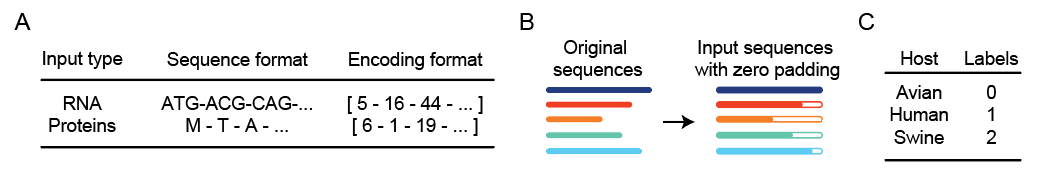


**Supplementary Figure 2. Data preprocessing.** (A) RNA and Protein sequences were encoded numerically using Index Based Encoding. (B) Input size is fixed using zero padding at the end of the sequences to ensure all input sequences have the same length. Input size is fixed based on maximal sequence length (PB2), i.e. 770 codons or amino acids. Representation of zero padding is shown with the hollowed sections. (C) Viral hosts are encoded by assigning each one a unique identifier.


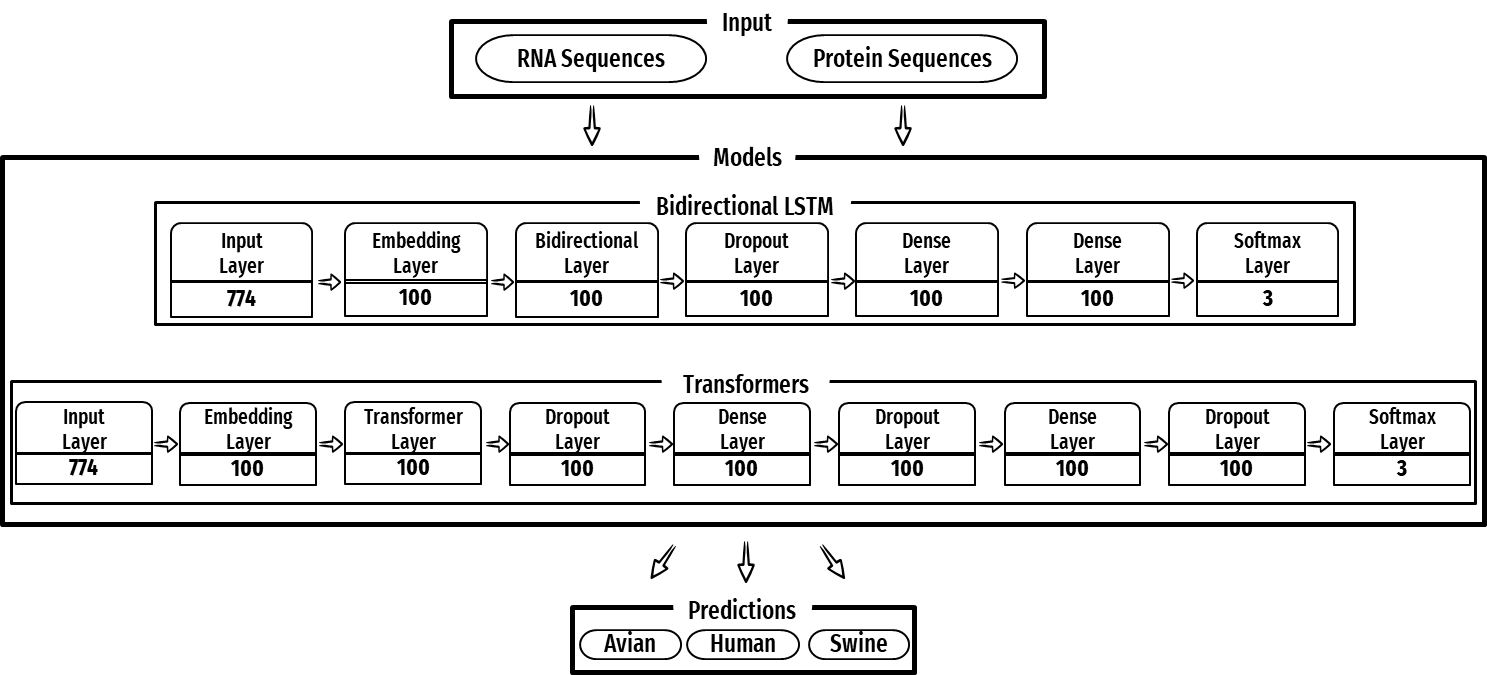


**Supplementary Figure 3. Deep learning architecture.** Bidirectional LSTMs and Transformers were used to predict viral hosts from either mRNA or protein viral sequences.


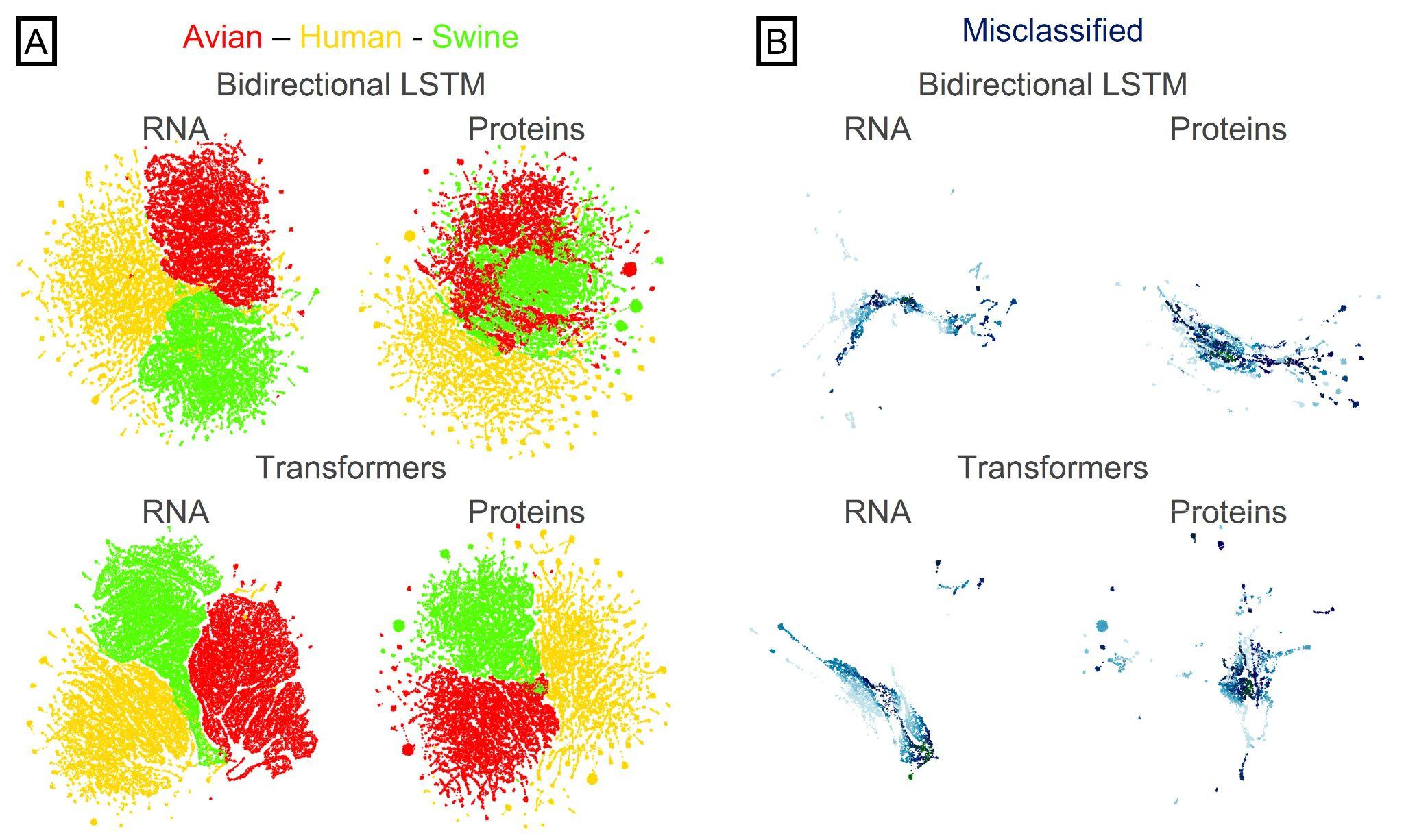


**Supplementary Figure 4. UMAP projection of latent spaces of the four deep learning models.** (A) UMAP visualization of well-classified sequences by hosts (red: avian, yellow: human, green: swine). (B) UMAP visualization of misclassified sequences in the same latent spaces. Opacity shows the degree of model inaccuracy. The darker the color, the more inaccurate the prediction, meaning that the network assigned a larger probability to an incorrect class. Each dot represents one gene from one viral strain.


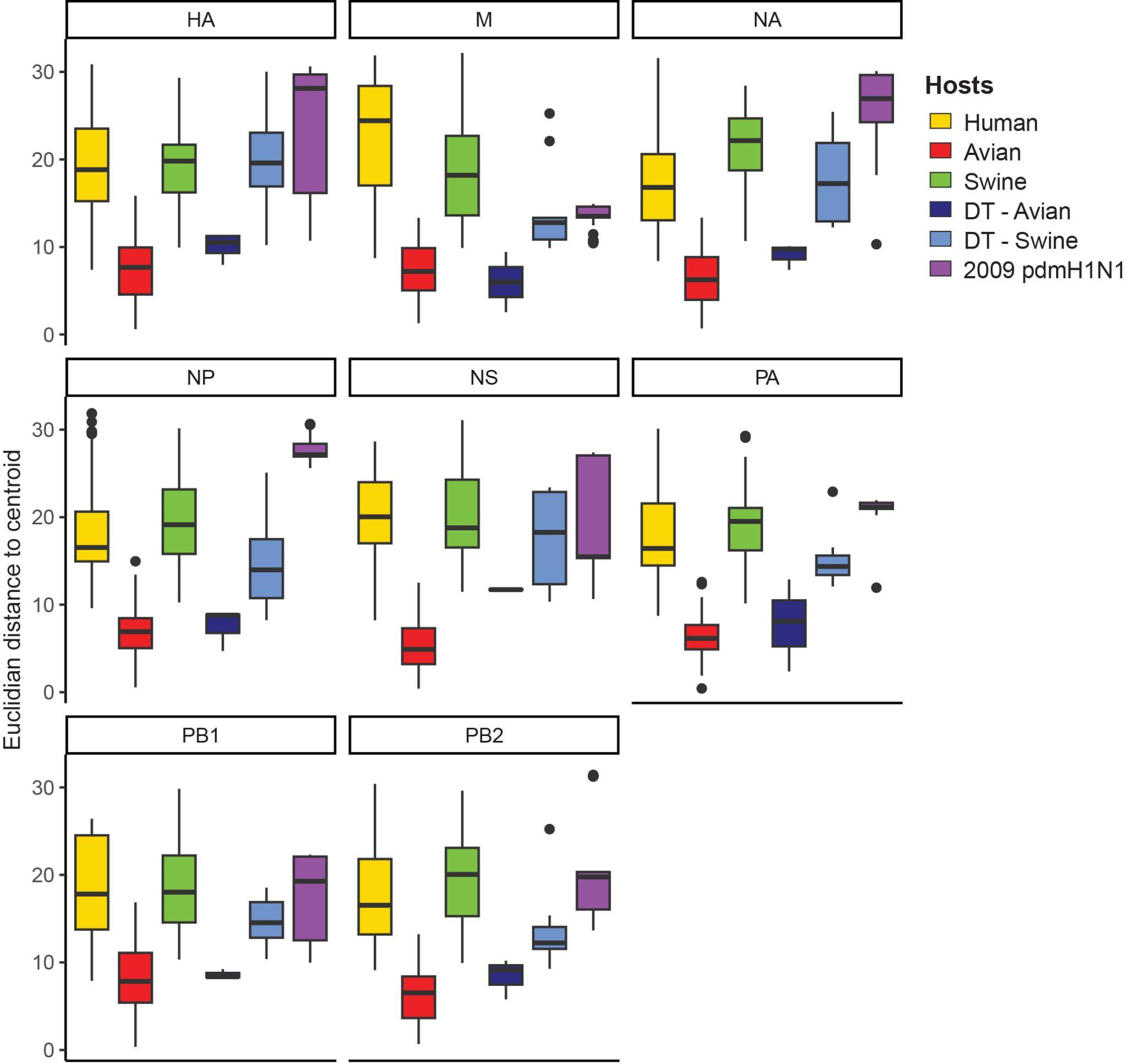


**Supplementary Figure 5A. Euclidean distance to centroids for different types of zoonotic sequences, per genes.** Euclidean distances to the cluster centroid in the latent space was calculated for each sequence. Results are shown separately for each viral gene. **(A) Distance to Avian centroid.**


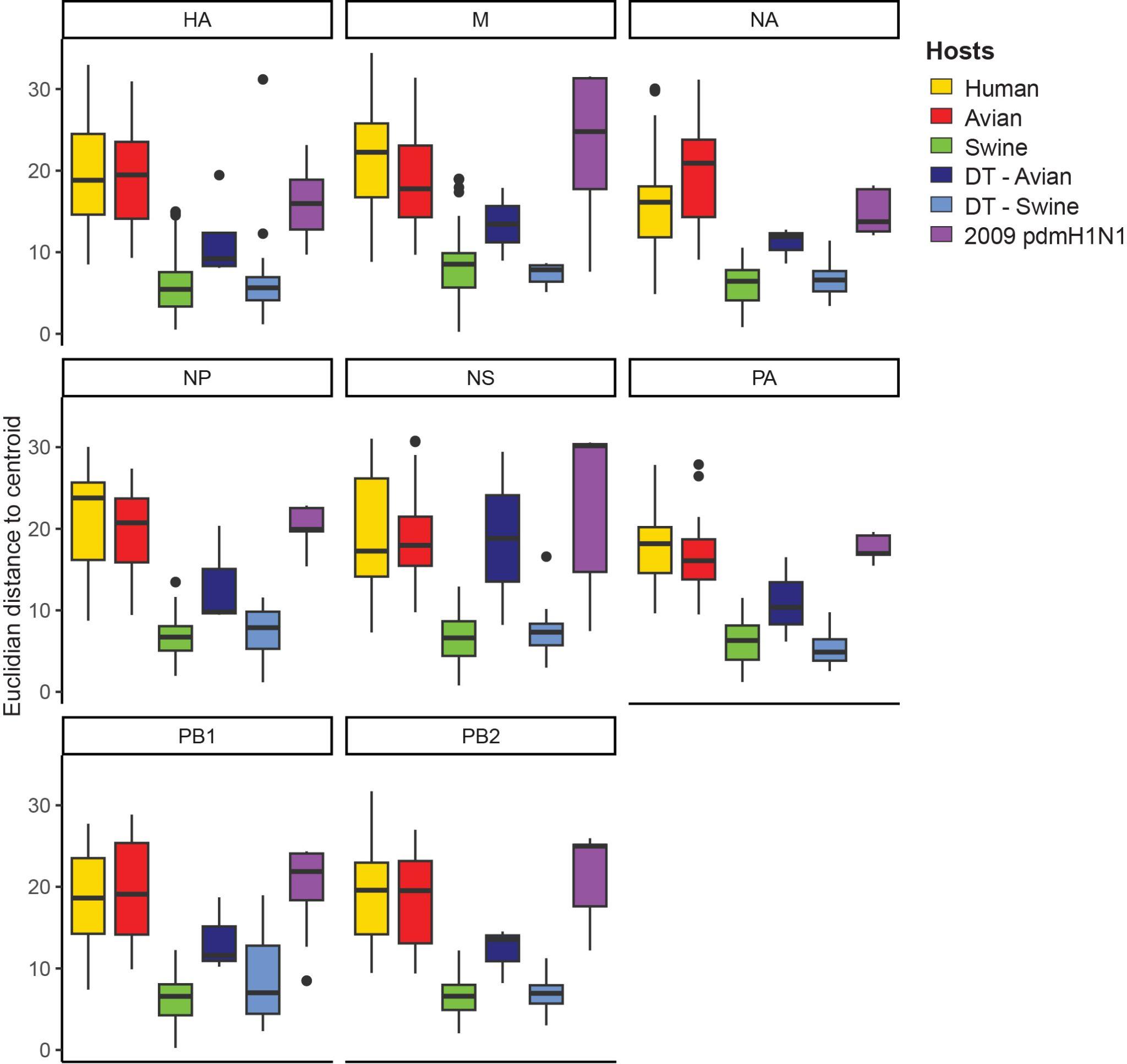


**Supplementary Figure 5B. Euclidean distance to centroids for different types of zoonotic sequences, per genes.** Euclidean distances to the cluster centroid in the latent space was calculated for each sequence. Results are shown separately for each viral gene. **(B) Distance to Swine centroid.**

**
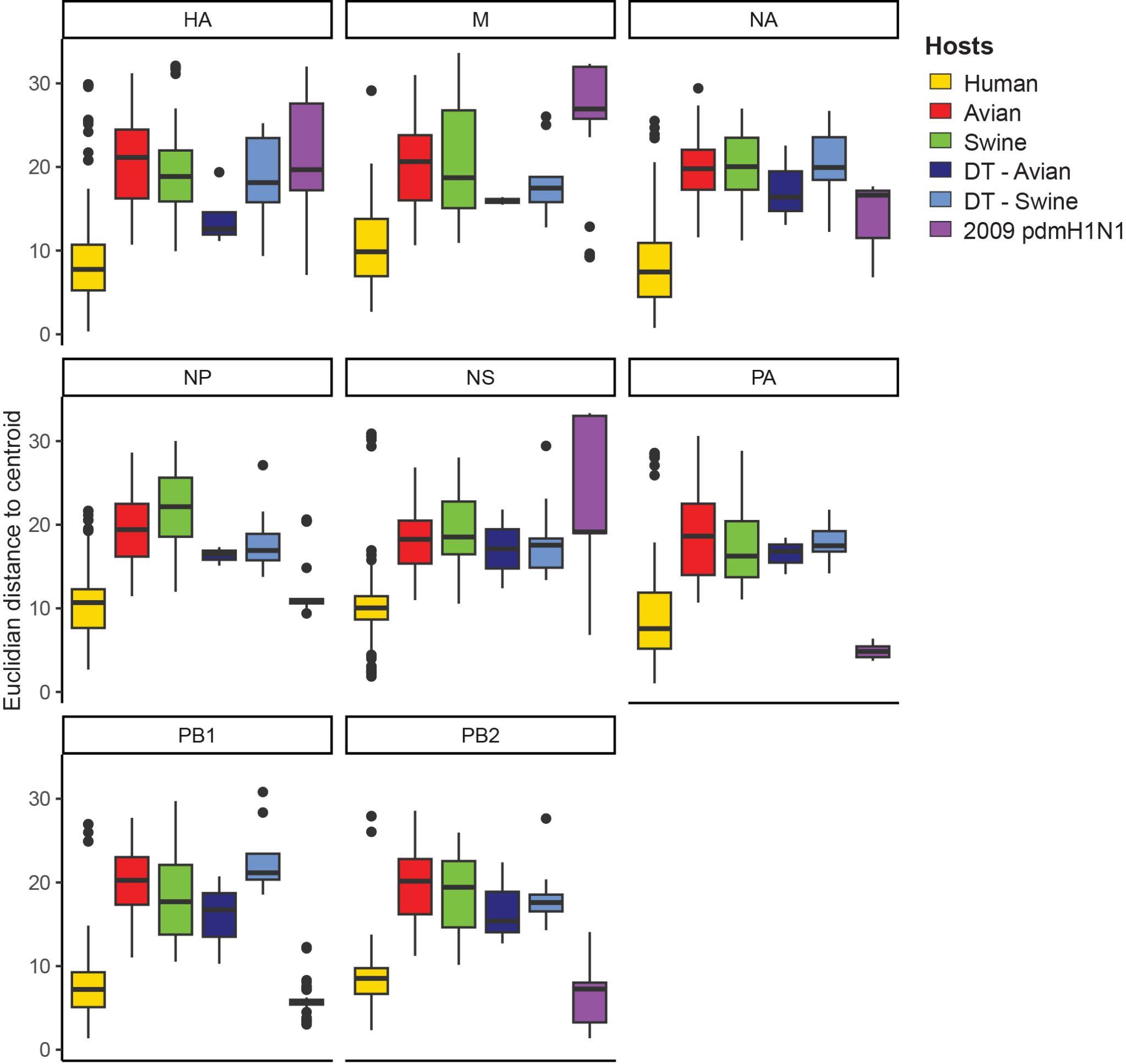
**

**Supplementary Figure 5C. Euclidean distance to centroids for different types of zoonotic sequences, per genes.** Euclidean distances to the cluster centroid in the latent space was calculated for each sequence. Results are shown separately for each viral gene. **(C) Distance to Human centroid.**


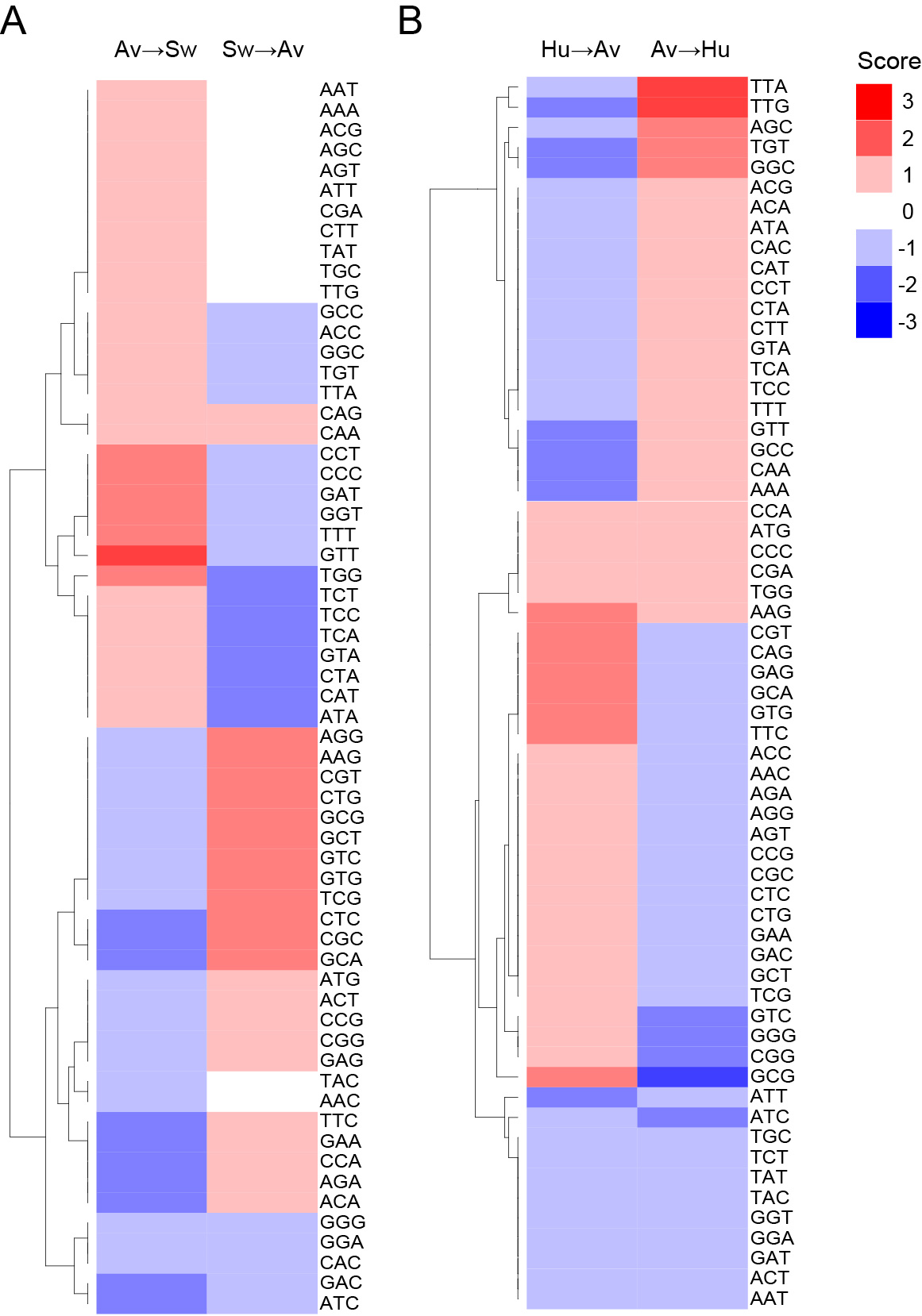


**Supplementary Figure 6. Codons likely to enhance or reduce zoonotic transition for Avian-Swine and Avian-Human pairs.** Codons were clustered for (A) Avian-Swine, or (B) for Avian-Human pairs, in both transition directions. Scores represent the number of methods which identified each codon as significantly enriched (positive sign) or depleted (negative sign) in misclassified sequences.

**
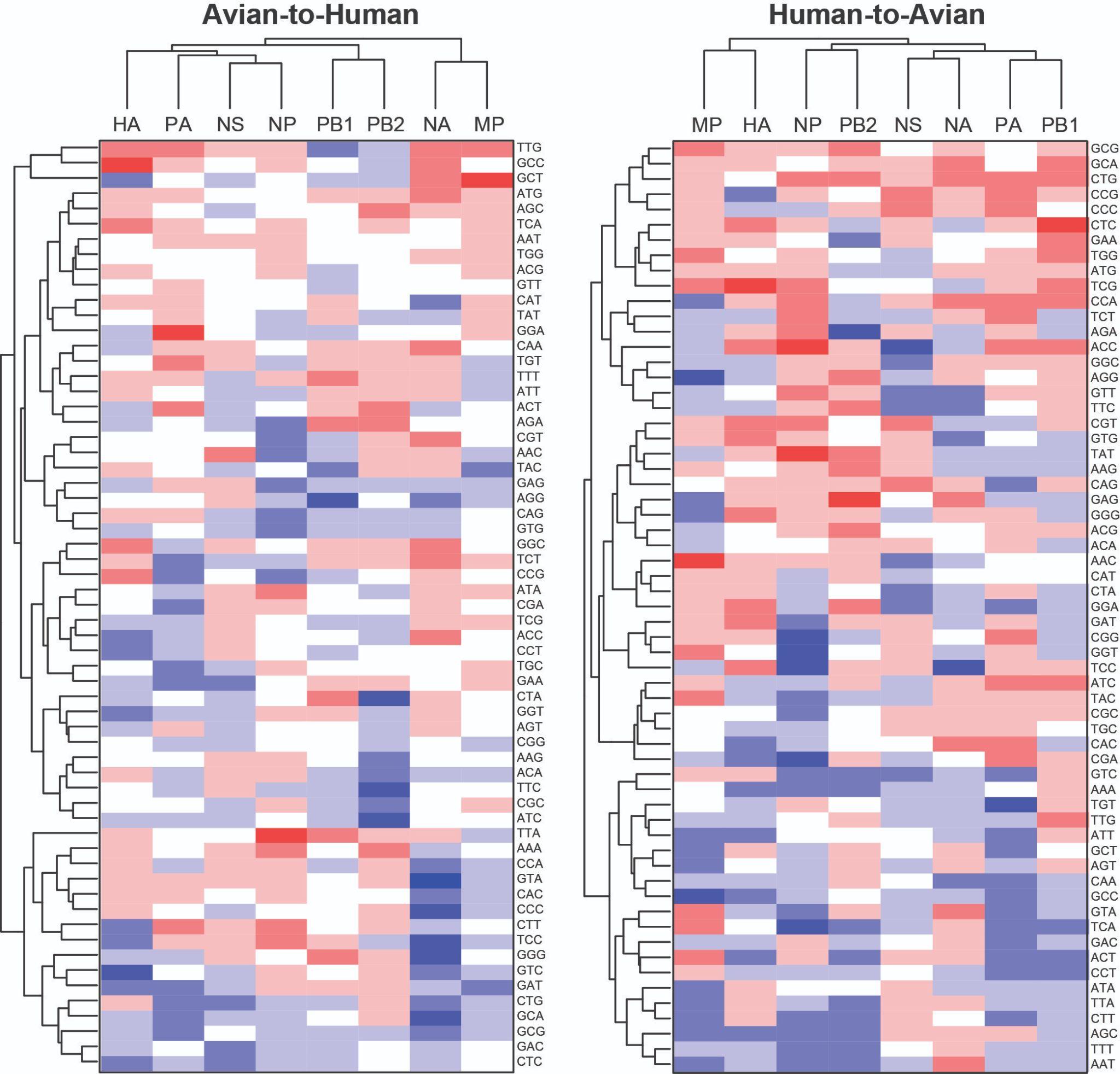
**

**Supplementary Figure 7. Codons likely to enhance or reduce zoonotic transition for each gene for Avian-Human pairs.** Codons were clustered for Avian-to-Human, or for Human-to-Avian sequences, in both transition directions. Scores represent the number of methods which identified each codon as significantly enriched (positive sign) or depleted (negative sign) in misclassified sequences.


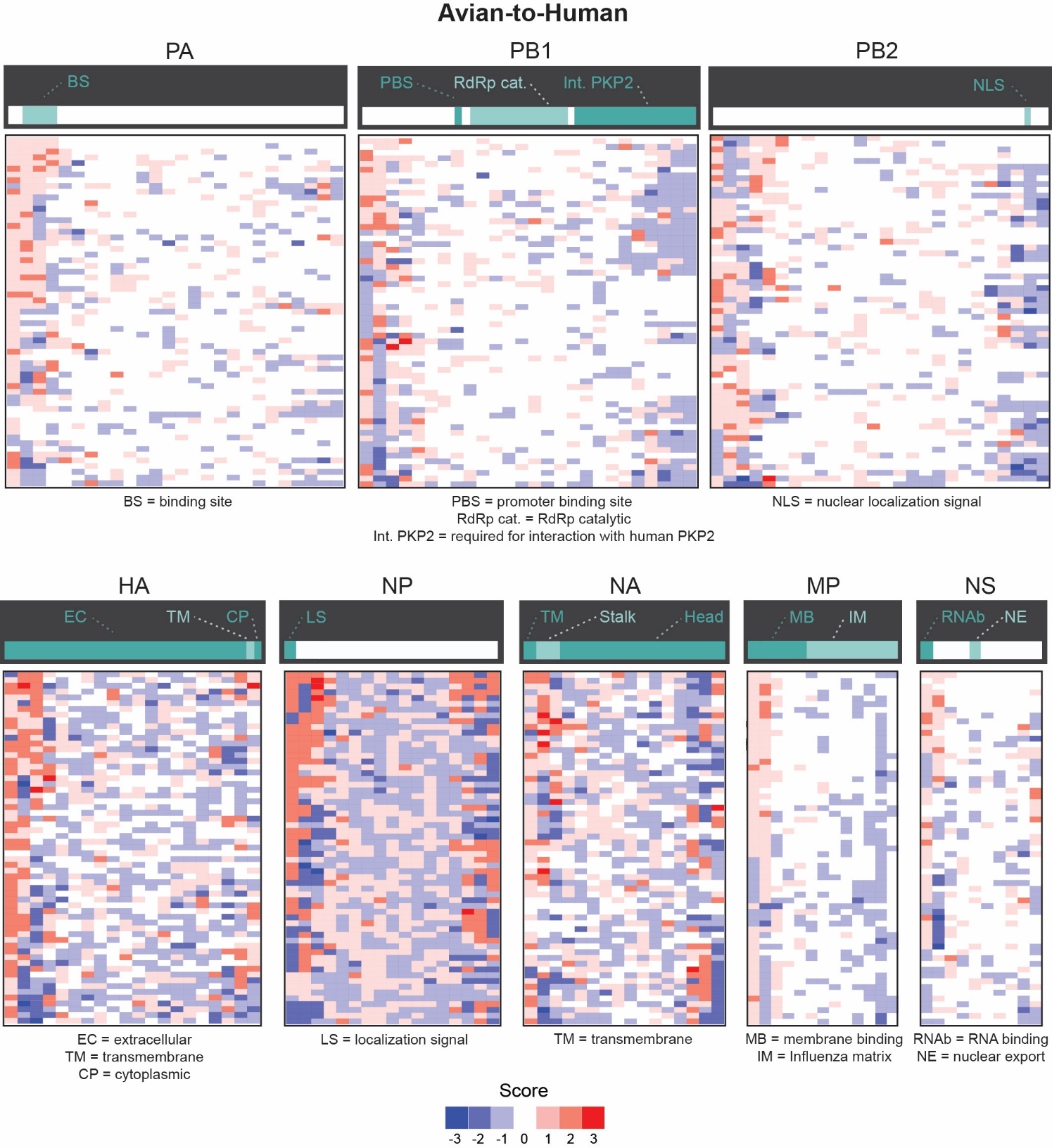


**Supplementary Figure 8.** **Candidate zoonotic transition biomarkers in Avian-to-Human misclassified sequences concentrate at the N’ and C’ ends of Influenza genes.** Codon features are analyzed separately for each section of 30 codons in each Influenza gene. Scores represent the number of methods which identified each codon as significantly enriched (positive sign) or depleted (negative sign) in misclassified sequences. Important gene domains are shown above in green shades.

## Supplementary Tables

**Supplementary Table 1. Hyperparameters of the models.**

| **Hyperparameter** | **Value** |
| --- | --- |
| Max epochs | 50 |
| Batch size | 32 |
| Loss | Categorical Cross Entropy |
| Optimizer | Adam |
| Last activation function | Softmax |
| Number of layers | Bidirectional LSTM: 7  Transformers: 9 |
| Size of layers | Input layer: 770  Hidden layers: 100  Output layer: 3 |

**Supplementary Table 2. Zoonotic strains with documented transmission from swine or avian to human, without subsequent human-to-human transmission.** All sequences were extracted from NCBI GenBank Database (n=85 sequences, from 13 strains originating from swine, and n=23 sequences from 2 strains originating from avian).

| **Origin** | **Virus name** | **Taxon** | **Virus type** | **Gene** | **Accession number** | **Reference** |
| --- | --- | --- | --- | --- | --- | --- |
| Avian | A/chicken/Lebanon/157/ 2016 | 1884996 | H5N1 | PB2  PB1  PA  HA  NP  NA  M  NS | KX644138  KX644139  KX644140  KX644141  KX644142  KX644143  KX644144  KX644145 | Schoch CL et al. Database 2020; baaa062 |
|  | A/goose/Guangdong/1/1996 | 93838 | H5N1 | NA  NA  HA  HA  HA  NP  NP  PA  PA  PB1  PB1  PB2  PB2  NS  M | NC_007361  AF144304  NC_007362  AF144305  AF148678  NC_007360  AF144303  NC_007359  AF144302  NC_007358  AF144301  NC_007357  AF144300  AF144307  AF144306 | Subbarao K, et al. Science. 1998; 279:393–396. |
| Swine | A/Hong Kong/1774/1999 | 231851 | H3N2 | PB2  PB1  PA  NA  NP  MP  HA  HA  HA  HA  HA  HA  HA  HA  NA  NA  NA  NA  MP  NS  NS  NS  NP  NP | AJ293920  AJ293921  AJ293922  AJ293923  AJ293924  AJ293925  AJ293926  AJ293927  AJ293928  AJ293929  AJ293930  AJ293931  AJ293932  AJ293933  AJ293934  AJ293935  AJ293936  AJ293937  AJ293938  AJ293939  AJ293940  AJ293941  AJ293942  AJ293943 | Garten RJ, et al. Science. 2009; 325(5937):197-201. |
| Swine | A/Thailand/271/2005 | 411157 | H1N1 | HA  MP  NS  NP  PB1  PB2  PA  NA | EF101749  EF101750  EF101751  EF101752  EF101753  EF101754  EF101755  EF101756 | Garten RJ, et al. Science. 2009; 325(5937):197-201. |
|  | A/Ohio/01/2007 | 643703 | H1N1 | HA | FJ986620 |  |
|  | A/Wisconsin/87/2005 | 643705 | H1N1 | HA | FJ986619 |  |
|  | A/Wisconsin/10/1998 | 157642 | H1N1 | NS  MP  NP  NA  HA  PA  PB1  PB2 | AF342817  AF342818  AF342819  AF342820  AF342821  AF342822  AF342823  AF342824 |  |
|  | A/Iowa/1/2006 | 643702 | H1N1 | HA | FJ986618 |  |
|  | A/Iowa/CEID23/2005 | 398800 | H1N1 | PB2  PB1  PA  NS  NP  NA  MP  HA | DQ889682  DQ889683  DQ889684  DQ889685  DQ889686  DQ889687  DQ889688  DQ889689 |  |
|  | A/Philippines/344/2004 | 411158 | H1N2 | HA  MP  NA  NS  NP  PA  PB2  PB1 | EF101741  EF101742  EF101743  EF101744  EF101745  EF101746  EF101747  EF101748 |  |
|  | A/Ohio/3559/1988 | 416743 | H1N1 | HA  MP  NA  NP  NS  PA  PB1  PB2 | CY024925  CY024926  CY024927  CY024928  CY024929  CY024930  CY024931  CY024932 |  |
|  | A/Wisconsin/301/1976 | 416742 | H1N1 | HA  MP  NA  NP  NS  PA  PB1  PB2 | CY178719  CY178720  CY178721  CY178722  CY178723  CY178724  CY178725  CY178726 |  |
| Swine | A/Michigan/09/2007 | 643706 | H1N2 | HA | FJ986622 | Shinde V. et al. NEJM 2009; 360 :2616-25 |
|  | A/Ohio/02/2007 | 643704 | H1N1 | HA | FJ986621 |  |
|  | A/South Korea/KS61/2016 | 11320 | H1N1 | M  NP  NA  NS  HA  PB1  PB2  PA | MH828297  MH828298  MH828299  MH828300  MH828301  MH828302  MH828303  MH828304 | Schoch CL et al. Database 2020; baaa062 |

**Supplementary Table 3. Zoonotic swine H1N1 strains from the 2009 pandemic.** All sequences were extracted from NCBI GenBank Database (n=476, from 77 strains). Accession numbers taken from Garten RJ et al, Science (2009).

| **Virus name** | **Taxon** | **Gene** | **Accession number** |
| --- | --- | --- | --- |
| A/ARIZONA/01/ 2009 | 643842 | HA  M  NA  NP  NS | GQ117067  GQ117066  GQ117064  GQ117063  GQ117065 |
| A/ARIZONA/02/ 2009 | 643843 | HA  M  NA  NP  PB1  PB2 | GQ117079  GQ117078  GQ117077  GQ117074  GQ117075  GQ117076 |
| A/CALIFORNIA/04/ 2009 | 641501 | HA  HA  HA  M  M  NA  NP  NP  NS  NS  PA  PA  PB1  PB2  PB2 | GQ117044  FJ966082  FJ966084  FJ969513  FJ966085  FJ969517  FJ969512  FJ966083  FJ969514  FJ966086  FJ969515  FJ966081  FJ966080  FJ969516  FJ966079 |
| A/CALIFORNIA/05/ 2009 | 641807 | HA  HA  M  NP  PA  PB1  PB2 | FJ966952  FJ966956  FJ966954  FJ966953  FJ966957  FJ966958  FJ966955 |
| A/CALIFORNIA/06/ 2009 | 641808 | HA  M  NA  NP  NS  PA  PB1  PB2 | FJ966960  FJ966962  FJ971075  FJ966961  FJ971074  FJ966964  FJ966965  FJ966963 |
| A/CALIFORNIA/07/ 2009 | 641809 | HA  HA  HA  M  M  M  NA  NP  NS  NS  PA  PA  PA  PB1  PB1  PB2  PB2  PB2 | FJ981613  FJ969540  FJ966974  FJ969527  FJ969537  FJ966975  FJ984386  FJ969536  FJ969528  FJ969538  FJ969529  FJ969539  FJ966977  FJ969531  FJ966978  FJ969530  FJ984387  FJ966976 |
| A/CALIFORNIA/08/ 2009 | 642261 | HA  M  M  NP  NS  NS  PA  PB1  PB2 | FJ971076  FJ969518  FJ969532  FJ984366  FJ969519  FJ969533  FJ984368  FJ984367  FJ984365 |
| A/CALIFORNIA/09/ 2009 | 641810 | HA  M  NA | FJ966971  FJ966972  FJ966973 |
| A/CALIFORNIA/10/ 2009 | 642262 | HA  M | FJ969511  FJ969510 |
| A/CALIFORNIA/11/ 2009 | 645258 | HA | GQ160579 |
| A/CALIFORNIA/12/ 2009 | 645270 | HA | GQ160606 |
| A/CALIFORNIA/13/ 2009 | 645271 | HA | GQ160607 |
| A/CALIFORNIA/14/ 2009 | 643844 | HA  M  NA  NP  NS  PA  PB1  PB2 | GQ117040  GQ117039  GQ117036  GQ117033  GQ117038  GQ117037  GQ117034  GQ117035 |
| A/COLORADO/03/2009 | 643845 | HA  NA  NP  PB1 | GQ117119  GQ117118  GQ117117  GQ168884 |
| A/INDIANA/09/ 2009 | 643846 | HA  M  NA  NP  NS  PA  PB1  PB2 | GQ117097  GQ117096  GQ117094  GQ117092  GQ168871  GQ117095  GQ117093  GQ168870 |
| A/MASSACHUSETTS/06/2009 | 643848 | HA  M  NA  NP  NS  PB1 | GQ117043  GQ168849  GQ117042  GQ117041  GQ168848  GQ168847 |
| A/MASSACHUSETTS /07/2009 | 643849 | HA  NA  NP  NS  PB1 | GQ117103  GQ117102  GQ117101  GQ168874  GQ168873 |
| A/MEXICO/3955/ 2009 | 645313 | HA  NA | GQ162194  GQ162193 |
| A/MEXICO/4108/ 2009 | 644882 | HA  HA  HA  HA  HA  HA  M  M  M  M  NA  NA  NA  NA  NA  NA  NP  NP  NP  NP  NS  NS  PA  PA  PA  PB1  PB2 | GQ149662  GQ149689  GQ149651  GQ149654  GQ149668  GQ162170  GQ149657  GQ149690  GQ149649  GQ162179  GQ149659  GQ149688  GQ149650  GQ149656  GQ149666  GQ162169  GQ149660  GQ149685  GQ149655  GQ149667  GQ149658  GQ149687  GQ149653  GQ149686  GQ149661  GQ149652  GQ162180 |
| A/MEXICO/4115/ 2009 | 644883 | HA  M  NA  NP  NS  PB1 | GQ149692  GQ149625  GQ149691  GQ149693  GQ149694  GQ162199 |
| A/MEXICO/4176/ 2009 | 645314 | HA  NA | GQ162182  GQ162181 |
| A/MEXICO/4269/ 2009 | 645315 | HA  NA | GQ162172  GQ162171 |
| A/MEXICO/4482/ 2009 | 644884 | HA  HA  HA  HA  M  M  NA  NA  NA  NP  NP  PA  PB1 | GQ149671  GQ149677  GQ162190  GQ149674  GQ149669  GQ162192  GQ149670  GQ162189  GQ149672  GQ149678  GQ149673  GQ149676  GQ149675 |
| A/MEXICO/4486/ 2009 | 644885 | HA  HA  HA  HA  HA  M  M  M  M  NA  NA  NA  NA  NA  NP  NP  NP  NS  NS  PA  PA  PB1  PB1  PB1  PB2  PB2 | GQ149647  GQ149623  GQ149665  GQ162185  GQ162202  GQ149642  GQ149624  GQ149663  GQ162188  GQ149644  GQ149622  GQ149664  GQ162187  GQ162201  GQ149645  GQ149619  GQ162184  GQ149643  GQ149621  GQ149646  GQ149620  GQ162176  GQ149618  GQ162186  GQ162177  GQ149617 |
| A/MEXICO/4502/ 2009 | 645316 | HA | GQ162195 |
| A/MEXICO/4516/ 2009 | 644886 | M  NP | GQ162178  GQ149648 |
| A/MEXICO/4575/ 2009 | 645317 | HA  NA | GQ162197  GQ162196 |
| A/MEXICO/4593/ 2009 | 645318 | HA  NA | GQ162174  GQ162173 |
| A/MEXICO/4595/ 2009 | 645319 | HA | GQ162191 |
| A/MEXICO/4603/ 2009 | 644887 | HA  HA  HA  M  M  M  NA  NA  NP  NP  NP  NS  NS  PA  PB1  PB1  PB2  PB2 | GQ149684  GQ149630  GQ149641  GQ149679  GQ149629  GQ162175  GQ149681  GQ149640  GQ149682  GQ149628  GQ149639  GQ149680  GQ149626  GQ149627  GQ149683  GQ162167  GQ162198  GQ162168 |
| A/MEXICO/4604/ 2009 | 644888 | HA  M  NA  NP  NS  PA  PB1  PB2 | GQ149634  GQ149638  GQ149631  GQ149637  GQ149635  GQ149636  GQ149633  GQ149632 |
| A/MEXICO/4627/ 2009 | 645320 | HA | GQ162183 |
| A/MEXICO/4635/ 2009 | 645321 | HA | GQ162200 |
| A/MEXICO/4646/ 2009 | 645322 | HA | GQ162204 |
| A/MICHIGAN/02/2009 | 643850 | HA  M  NA  NP  NS  PA  PB1 | GQ117112  GQ117111  GQ117108  GQ117107  GQ117110  GQ117109  GQ168880 |
| A/MINNESOTA/ 02/2009 | 643851 | M  NA  NP  NS  PB1  PB2 | GQ117073  GQ117071  GQ117068  GQ117072  GQ117069  GQ117070 |
| A/NEBRASKA/02/2009 | 643852 | NA  NP  NS  PB1 | GQ117105  GQ117104  GQ117106  GQ168875 |
| A/NEBRASKA/03/2009 | 645265 | HA  NP  NS  PB1  PB2 | GQ160599  GQ160595  GQ160598  GQ160596  GQ160597 |
| A/NEW YORK/04/ 2009 | 645255 | HA | GQ160568 |
| A/NEW YORK/05/ 2009 | 645247 | HA | GQ160542 |
| A/NEW YORK/06/ 2009 | 643540 | HA  M  NA  NP  NS  PB1 | GQ168851  FJ984338  FJ984340  FJ984341  FJ984339  GQ168850 |
| A/NEW YORK/08/ 2009 | 645735 | HA  M  NA | GQ168650  GQ168649  GQ168648 |
| A/NEW YORK/09/ 2009 | 643853 | HA  HA  NA  PB1 | GQ168655  GQ168886  GQ168654  GQ117120 |
| A/NEW YORK/10/ 2009 | 643541 | HA  M  NA  NP  NS  PA  PB1  PB2 | FJ984375  FJ984369  FJ984371  FJ984372  FJ984370  FJ984374  FJ984373  GQ168876 |
| A/NEW YORK/11/ 2009 | 643542 | HA  HA  M  NA  NA  NP  NS  PA  PB1 | FJ984347  GQ168652  FJ984342  FJ984344  GQ168651  FJ984345  FJ984343  FJ984346  GQ168852 |
| A/NEW YORK/12/ 2009 | 643543 | HA  HA  M  NA  NA  NP  NS  PB1 | FJ984337  GQ168673  GQ168845  FJ984335  GQ168672  FJ984336  FJ984334  GQ168844 |
| A/NEW YORK/13/ 2009 | 643854 | HA  HA  M  M  NA  NA  NP  NS  PA  PB1 | GQ117116  GQ168668  GQ168883  GQ168667  GQ117114  GQ168666  GQ117113  GQ168882  GQ117115  GQ168881 |
| A/NEW YORK/14/ 2009 | 645737 | HA  NA | GQ168657  GQ168656 |
| A/NEW YORK/15/ 2009 | 643544 | M  NA  NP  NS  PA  PB1  PB2 | FJ984376  FJ984378  FJ984379  FJ984377  FJ984380  GQ168877  GQ168878 |
| A/NEW YORK/18/ 2009 | 643545 | HA  HA  M  NA  NP  NS  PA  PB1  PB2 | FJ984355  GQ160556  FJ984348  FJ984350  FJ984352  FJ984349  FJ984354  FJ984353  FJ984351 |
| A/NEW YORK/19/ 2009 | 642264 | HA  HA  M  NA  NP  NS  PA  PB1 | FJ984394  FJ969509  FJ984388  FJ984390  FJ984391  FJ984389  FJ984393  FJ984392 |
| A/NEW YORK/20/ 2009 | 642265 | HA  HA  HA  M  M  NA  NA  NA  NP  NP  NS  NS  PB1 | GQ117082  GQ117086  FJ969542  GQ168863  GQ117085  GQ117081  GQ117084  FJ969541  GQ117080  GQ117083  GQ168862  GQ168868  GQ168867 |
| A/NEW YORK/22/ 2009 | 643855 | HA  M  NA  NP  NS  PB1  PB2 | GQ117024  GQ117023  GQ117022  GQ117019  GQ168846  GQ117020  GQ117021 |
| A/NEW YORK/23/ 2009 | 643546 | HA  HA  M  NA  NA  NP  NS  PA  PB1 | FJ984364  GQ168628  GQ168866  FJ984362  GQ168627  FJ984363  FJ984361  GQ168865  GQ168864 |
| A/NEW YORK/24/ 2009 | 645266 | HA  M  NA | GQ160601  GQ160600  GQ168669 |
| A/NEW YORK/26/ 2009 | 645725 | HA | GQ168617 |
| A/NEW YORK/27/ 2009 | 645738 | HA  NA | GQ168659  GQ168658 |
| A/NEW YORK/28/ 2009 | 645733 | HA | GQ168645 |
| A/NEW YORK/30/ 2009 | 645253 | HA | GQ160566 |
| A/NEW YORK/31/ 2009 | 643547 | HA  HA  M  NA  NA  NP  NS  PA | FJ984360  GQ168661  GQ168859  FJ984357  GQ168660  FJ984358  FJ984356  FJ984359 |
| A/NEW YORK/34/ 2009 | 645254 | HA | GQ160567 |
| A/NEW YORK/35/ 2009 | 645248 | HA | GQ160543 |
| A/NEW YORK/37/ 2009 | 645234 | HA | GQ160527 |
| A/NEW YORK/39/ 2009 | 645267 | HA | GQ160602 |
| A/OHIO/07/2009 | 642266 | HA  HA  HA  HA  HA  M  M  NA  NA  NA  NP  NP  NS  NS  PA  PB1  PB1  PB2 | FJ984401  GQ117100  FJ984397  FJ969521  FJ969535  FJ984398  FJ984395  GQ117099  FJ969520  FJ969534  GQ117098  FJ984396  FJ984399  GQ168872  FJ984400  GQ160544  GQ168887  GQ160545 |
| A/SOUTH CAROLINA/09/ 2009 | 643856 | HA  M  NA  NP  NS  PB1 | GQ117056  GQ117055  GQ117053  GQ117052  GQ117054  GQ168854 |
| A/TEXAS/04/2009 | 641811 | HA  HA  HA  M  M  M  NA  NA  NP  NP  NS  PA  PA  PB1  PB1  PB2  PB2 | FJ981615  FJ981612  FJ966982  FJ981617  FJ966980  FJ966983  FJ981614  FJ966981  FJ981618  FJ966979  FJ981620  FJ981619  FJ969524  FJ981616  FJ969526  GQ168885  FJ969525 |
| A/TEXAS/05/2009 | 641812 | HA  HA  M  M  NA  NP  NP  NS  NS  PA  PA  PB1  PB1 | GQ168861  FJ966959  FJ966968  FJ981608  FJ966969  FJ966967  FJ981609  FJ966966  FJ981611  FJ966970  FJ981610  FJ969522  GQ168860 |
| A/TEXAS/06/2009 | 643548 | HA  M  NA  NP  NS  PB2 | FJ984385  FJ984381  FJ984383  FJ984384  FJ984382  GQ168879 |
| A/TEXAS/07/2009 | 643857 | HA  M  NP  NS  PB1  PB2 | GQ117091  GQ117090  GQ117087  GQ168869  GQ117088  GQ117089 |
| A/TEXAS/08/2009 | 643858 | HA  HA  M  NA  NA  NP  NS  PA  PB1  PB2 | GQ117051  GQ168633  GQ117050  GQ117048  GQ168632  GQ117045  GQ168853  GQ117049  GQ117046  GQ117047 |
| A/TEXAS/09/2009 | 643859 | HA  HA  M  NA  NA  NP  NS  PA  PB1  PB2 | GQ117032  GQ168671  GQ117031  GQ117028  GQ168670  GQ117025  GQ117030  GQ117029  GQ117026  GQ117027 |
| A/TEXAS/15/2009 | 644199 | HA  M  NA  NP  NS  PB1 | GQ122097  GQ122095  GQ122096  GQ122092  GQ122093  GQ122094 |
| A/TEXAS/19/2009 | 645726 | HA  M  NP | GQ168620  GQ168619  GQ168618 |
| A/TEXAS/22/2009 | 645256 | HA  M  NA  NP  NS  PB1 | GQ160574  GQ160573  GQ160571  GQ160569  GQ160572  GQ160570 |
| A/TEXAS/23/2009 | 645249 | HA  M  NA  NS  PB1 | GQ160550  GQ160549  GQ160547  GQ160548  GQ160546 |

**Supplementary Table 4. Accuracy of host classification models on train, validation and test sets.**

|  | **Bidirectional LSTM** | | | **Transformers** | | |
| --- | --- | --- | --- | --- | --- | --- |
|  | Train | Validation | Test | Train | Validation | Test |
| **RNA** | 0.9600 | 0.9566 | **0.9565** | 0.9570 | 0.9517 | **0.9511** |
| **Proteins** | 0.9352 | 0.9329 | 0.9333 | 0.9387 | 0.9358 | 0.9352 |

**Supplementary Table 5. Proportion of well-classified and misclassified sequences for each host.**

|  | RNA | | Proteins | |
| --- | --- | --- | --- | --- |
|  | Bidirectional LSTM | Transformers | Bidirectional LSTM | Transformers |
| Avian  Avian_to_Human  Avian_to_Swine | 99.1%  0.3%  0.6% | 97.5%  0.9%  1.6% | 98.1%  0.6%  1.3% | 98.6%  0.9%  0.5% |
| Human  Human_to_Avian  Human_to_Swine | 95.6%  1.4%  3.0% | 97.0%  1.3%  1.7% | 91.5%  1.6%  6.8% | 94.8%  2.3%  2.9% |
| Swine  Swine_to_Avian  Swine_to_Human | 92.0%  1.6%  6.3% | 90.8%  1.4%  7.8% | 90.2%  2.9%  6.9% | 86.9%  2.9%  10.2% |

**Supplementary Table 6. Codon usage differences in misclassified sequences by pairs of species.**

| **AA** | **Codons** | **Sw_Hu** | **Hu_Sw** | **Av_Hu** | **Hu_Av** | **Sw_Av** | **Av_Sw** |
| --- | --- | --- | --- | --- | --- | --- | --- |
| Ala | GCA | 1 | 1 | -1 | **2** | 2 | -2 |
|  | GCC | 0 | 2 | 1 | **-2** | -1 | 1 |
|  | GCG | 0 | 2 | -3 | **2** | 2 | -1 |
|  | GCT | -1 | 3 | -1 | 1 | 2 | -1 |
| Arg | AGA | 1 | -1 | -1 | 1 | 1 | -2 |
|  | AGG | -1 | -1 | -1 | 1 | 2 | -1 |
|  | CGA | -1 | **2** | 1 | 1 | 0 | 1 |
|  | CGG | 0 | **-2** | -2 | 1 | 1 | -1 |
|  | CGC | -2 | **2** | -1 | 1 | 2 | -2 |
|  | CGT | -2 | 1 | -1 | 2 | 2 | -1 |
| Asn | AAC | 1 | -1 | -1 | 1 | 0 | -1 |
|  | AAT | 1 | -1 | -1 | -1 | 0 | 1 |
| Asp | GAC | 1 | -1 | -1 | 1 | -1 | **-2** |
|  | GAT | -1 | -1 | -1 | -1 | -1 | **2** |
| Cys | TGC | -1 | -1 | -1 | -1 | 0 | 1 |
|  | TGT | 1 | -1 | 2 | -2 | -1 | 1 |
| Gln | CAA | -1 | **-2** | 1 | **-2** | 1 | 1 |
|  | CAG | 0 | **3** | -1 | **2** | 1 | 1 |
| Glu | GAA | 1 | 1 | -1 | 1 | 1 | -2 |
|  | GAG | 0 | 1 | -1 | 2 | 1 | -1 |
| Gly | GGA | -1 | -1 | -1 | -1 | -1 | -1 |
|  | GGC | 1 | -1 | **2** | -2 | -1 | 1 |
|  | GGG | 1 | -1 | **-2** | 1 | -1 | -1 |
|  | GGT | -1 | 1 | -1 | -1 | -1 | 2 |
| His | CAC | 1 | -1 | 1 | -1 | -1 | -1 |
|  | CAT | -1 | 1 | 1 | -1 | -2 | 1 |
| Ile | ATA | 2 | -2 | 1 | -1 | -2 | 1 |
|  | ATC | -1 | 1 | -2 | -1 | -1 | -2 |
|  | ATT | 1 | -1 | -1 | -2 | 0 | 1 |
| Leu | CTA | 1 | 3 | 1 | -1 | **-2** | 1 |
|  | CTC | -1 | 2 | -1 | 1 | **2** | -2 |
|  | TTA | 1 | -1 | 3 | -1 | -1 | 1 |
|  | TTG | 0 | -1 | 3 | -2 | 0 | 1 |
|  | CTG | -2 | 1 | -1 | 1 | **2** | -1 |
|  | CTT | -2 | 1 | 1 | -1 | 0 | 1 |
| Lys | AAA | 1 | -2 | 1 | **-2** | 0 | 1 |
|  | AAG | 2 | 1 | 1 | **2** | 2 | -1 |
| Met | ATG | -1 | 1 | 1 | 1 | 1 | -1 |
| Phe | TTC | 1 | -1 | -1 | 2 | 1 | **-2** |
|  | TTT | -1 | -1 | 1 | -1 | -1 | **2** |
| Pro | CCA | 1 | -1 | 1 | 1 | 1 | **-2** |
|  | CCC | -1 | 1 | 1 | 1 | -1 | **2** |
|  | CCG | 2 | 2 | -1 | 1 | 1 | -1 |
|  | CCT | -1 | -1 | 1 | -1 | -1 | **2** |
| Ser | AGT | -2 | 1 | -1 | 1 | 0 | 1 |
|  | AGC | -1 | -1 | 2 | -1 | 0 | 1 |
|  | TCA | 1 | -1 | 1 | -1 | **-2** | 1 |
|  | TCG | -1 | 2 | -1 | 1 | **2** | -1 |
|  | TCT | 1 | -1 | -1 | -1 | **-2** | 1 |
|  | TCC | -2 | -1 | 1 | -1 | **-2** | 1 |
| Thr | ACC | **-3** | 1 | -1 | 1 | -1 | 1 |
|  | ACA | **2** | -1 | 1 | -1 | 1 | -2 |
|  | ACG | -1 | 2 | 1 | -1 | 0 | 1 |
|  | ACT | -1 | 1 | -1 | -1 | 1 | -1 |
| Trp | TGG | 1 | -1 | 1 | 1 | -2 | 2 |
| Tyr | TAC | 1 | 1 | -1 | -1 | 0 | -1 |
|  | TAT | 1 | -1 | -1 | -1 | 0 | 1 |
| Val | GTG | **-2** | 1 | -1 | **2** | **2** | -1 |
|  | GTA | **2** | -1 | 1 | -1 | **-2** | 1 |
|  | GTC | **2** | 1 | -2 | 1 | **2** | -1 |
|  | GTT | -1 | -1 | 1 | **-2** | -1 | 3 |
